# Understanding Adolescent and Young Adult 6-Mercaptopurine Adherence and mHealth Engagement During Cancer Treatment: Protocol for Ecological Momentary Assessment

**DOI:** 10.2196/32789

**Published:** 2021-10-22

**Authors:** Alexandra M Psihogios, Mashfiqui Rabbi, Annisa Ahmed, Elise R McKelvey, Yimei Li, Jean-Philippe Laurenceau, Stephen P Hunger, Linda Fleisher, Ahna LH Pai, Lisa A Schwartz, Susan A Murphy, Lamia P Barakat

**Affiliations:** 1 Children's Hospital of Philadelphia University of Pennsylvania Philadelphia, PA United States; 2 Department of Statistics Harvard University Boston, MA United States; 3 Division of Oncology Children's Hospital of Philadelphia Philadelphia, PA United States; 4 Children's Hospital of Philadelphia La Salle University Philadelphia, PA United States; 5 Department of Psychology University of Delaware Newark, DE United States; 6 Health Communications and Health Disparities Fox Chase Cancer Center Philadelphia, PA United States; 7 Cincinnati Children's Hospital Medical Center University of Cincinnati College of Medicine Cincinnati, OH United States

**Keywords:** mHealth, ecological momentary assessment, adolescents, young adults, oncology, cancer, self-management, mobile phone

## Abstract

**Background:**

Adolescents and young adults (AYAs) with cancer demonstrate suboptimal oral chemotherapy adherence, increasing their risk of cancer relapse. It is unclear how everyday time-varying contextual factors (eg, mood) affect their adherence, stalling the development of personalized mobile health (mHealth) interventions. Poor engagement is also a challenge across mHealth trials; an effective adherence intervention must be engaging to promote uptake.

**Objective:**

This protocol aims to determine the temporal associations between daily contextual factors and 6-mercaptopurine (6-MP) adherence and explore the proximal impact of various engagement strategies on ecological momentary assessment survey completion.

**Methods:**

At the Children’s Hospital of Philadelphia, AYAs with acute lymphoblastic leukemia or lymphoma who are prescribed prolonged maintenance chemotherapy that includes daily oral 6-MP are eligible, along with their matched caregivers. Participants will use an ecological momentary assessment app called ADAPTS (Adherence Assessments and Personalized Timely Support)—a version of an open-source app that was modified for AYAs with cancer through a user-centered process—and complete surveys in bursts over 6 months. Theory-informed engagement strategies will be microrandomized to estimate the causal effects on proximal survey completion.

**Results:**

With funding from the National Cancer Institute and institutional review board approval, of the proposed 30 AYA-caregiver dyads, 60% (18/30) have been enrolled; of the 18 enrolled, 15 (83%) have completed the study so far.

**Conclusions:**

This protocol represents an important first step toward prescreening tailoring variables and engagement components for a just-in-time adaptive intervention designed to promote both 6-MP adherence and mHealth engagement.

**International Registered Report Identifier (IRRID):**

DERR1-10.2196/32789

## Introduction

### Background

For patients with acute lymphoblastic leukemia or lymphoma, durable cancer remission requires a prolonged maintenance phase characterized by 18-30 months of a regimen that includes daily oral intake of the chemotherapy agent 6-mercaptopurine (6-MP) [1,2]. In a trial conducted by the Children’s Oncology Group, nearly 50% of children and adolescents demonstrated electronically monitored 6-MP adherence rates below a 95% critical level for relapse prevention, resulting in a 2.5 times greater risk for relapse in nonadherent patients [1]. In this study and others, adolescents and young adults (AYAs) with cancer demonstrated lower oral chemotherapy adherence than their younger counterparts [2-6]. AYA treatment adherence is often at odds with normative developmental goals, such as establishing autonomy and navigating social pressures, as well as neurodevelopmental changes that occur during these years (eg, developing executive functions) [7-9]. Although cross-sectional studies have begun to identify contextual risk factors for oral chemotherapy nonadherence, including physical symptoms [10,11], negative mood [10,12], low motivation [13,14], difficult family interactions [15-17], and environmental factors (eg, being outside of the home) [15,17,18], these multifactorial and dynamic contexts can vary from day to day within individuals [19]. It remains unclear how these idiosyncratic factors affect daily 6-MP adherence, complicating the development of personalized mobile health (mHealth) interventions to deliver effective, contextualized, and timely adherence support [20-23]. For addressing this gap, this paper describes the protocol for an app-based ecological momentary assessment (EMA) study of AYAs with cancer.

EMA involves frequent surveys about behaviors and experiences in real time, often via SMS text messaging or mobile apps [24-26], making it a particularly appealing methodology for native smartphone users such as AYAs. EMA offers several distinct advantages for advancing adherence science by reducing recall bias, increasing real-world generalizability, and providing multilevel data on how adherence behaviors play out over time, place, and context [27]. In a pilot study, we demonstrated that implementing daily EMA for 6-MP adherence, across 28 days, was feasible and acceptable for AYAs with leukemia [28]. AYAs were more likely to miss a dose of 6-MP on weekends and on days when their adherence motivation and negative affect were worse relative to their typical functioning. Although these findings were novel and offered potential decision rules to test in future trials (eg, *deliver a medication reminder on weekends when AYA may be more prone to forget*), they require replication with a larger sample, over a longer period of time, and including AYA’s caregivers who are often intricately involved in cancer medication management (in one study, 73% of caregivers were responsible for managing these medications) [17].

A challenge with EMA and mHealth tools, in general, is rapidly declining user engagement [29-32]. All the methodological advantages of EMA are weakened if participants miss substantial surveys or stop responding altogether [29]. Moreover, AYAs with the greatest treatment adherence challenges—who would stand to benefit the most from an adherence-promotion intervention—may be the least likely to engage with EMA [28,33]. Although our pilot study demonstrated relatively high and stable EMA survey completion rates (mean 88.9%, SD 16.7%), there was substantial variability (range 39.3%-100%), and users were compensated US $2 for completing all 14 survey questions per day (up to US $56 total across 28 days) [28]. Financial incentives are an effective method to promote mHealth engagement in the short term; however, evidence supporting their long-term efficacy is mixed, and they may not be scalable in real-world clinical settings [34].

Just-in-time adaptive interventions (JITAIs) use tailoring variables (eg, information about a participant used to make decisions, such as perceptions of mood, motivation, and family interactions) and decision rules (eg, specifying which intervention to offer and to whom, based on the tailoring variable) to deliver interventions adapted to the participant’s internal and external states [35]. At this time, many of the hypothesized contextual tailoring variables related to 6-MP adherence (eg, motivation and family functioning) can only be collected via EMA (rather via sensors). Thus, identifying lower cost and effective EMA engagement strategies is an essential prerequisite for the design of clinical trials that optimize adherence-promotion interventions and even eventual real-world implementations of strategies identified to be effective.

Various fields, including psychology, human-computer interaction, and marketing, highlight alternative strategies for engaging individuals. Engagement strategies may include social influence tactics (eg, targeting reciprocity by providing a *no strings attached* reward to increase the likelihood of later completing a survey) and operant conditioning behavioral principles (eg, receiving a desirable reward that reinforces survey completion) [36]. Rewards may facilitate extrinsic motivation (eg, completing a survey as it results in something immediately gratifying, like a funny meme) or intrinsic motivation (eg, completing a survey as the data will help someone else) [37]. To date, limited research has focused on optimizing such engagement strategies in mHealth tools, where the ultimate goal is to dynamically adapt engagement components to match the unique characteristics and changing responses of users [36].

### Objectives

In this study, we adopted and refined an open-source EMA app called SARA [38] for an AYA cancer population (renamed ADAPTS [Adherence Assessments and Personalized Timely Support]). Through using this app, our primary study aim is to determine the temporal associations between daily contextual factors and 6-MP adherence. We draw on the pediatric self-management model [39], a social-ecological theoretical framework of disease self-management and treatment adherence, focusing primarily on the individual-and family-level contexts that could affect day-to-day medication adherence. Specifically, we hypothesize that daily fluctuations in intrapersonal (eg, physical symptoms, negative mood, and low motivation) and interpersonal or environmental (eg, a dyadic AYA-caregiver disagreement and being outside of the home without caregivers) factors will increase the odds of missing 6-MP that day. In a secondary exploratory aim, we will explore the proximal impacts of theory-informed engagement strategies (reciprocity and nonmonetary reinforcements) on daily EMA survey completion. Our approach is consistent with the first step of the Behavior Change Theories, User-Centered Design, and Social Marketing framework, a comprehensive model for creating mHealth tools, which recommends conducting a situational analysis of the contextual factors associated with a health problem [40], as well as the preparation phase of the multiphase optimization strategy [41] comprised of gathering initial information to decide which set of (engagement) components to include before an optimization trial. These formative data will ultimately inform the creation of the first JITAI for improving AYA oral chemotherapy adherence while running a concurrent JITAI for promoting EMA engagement. Improving the precision of adherence-promotion interventions in this manner may significantly benefit AYAs who require frequent self-administration of medications, as static interventions have yielded small and heterogeneous effects [42,43].

## Methods

### Study Design

This study uses an intensive longitudinal burst design to periodically deliver EMA surveys over 6 months of maintenance chemotherapy that includes daily prescribed oral 6-MP to AYAs with acute lymphoblastic leukemia or lymphoma and their caregivers. Within the observational EMA study is an embedded microrandomized trial—an experimental design for optimizing mHealth interventions [29]—that randomizes engagement components within person and across EMAs as an early check on their efficacy before incorporating and piloting in a later JITAI.

At baseline, the enrolled AYAs and their caregivers will complete a demographic survey; brief screening measure of executive functioning; and assessment of COVID-19 exposure, impact, and distress [44] via REDCap (Research Electronic Data Capture; Vanderbilt University). Using the ADAPTS app, each participant will then receive EMA in *bursts* over the course of 6 months of maintenance therapy, capturing a time frame known to be associated with AYA 6-MP nonadherence and cancer relapse. In month 1 (28 days; ie, burst 1), EMA surveys will be sent once per day via the app to fully capture contextual processes during the month between follow-up medical appointments. To balance participant burden while also ensuring data are representative, in months 2-6, EMA surveys will be delivered once per day at the same time (6 PM), for one full week (2 weeks after the last maintenance appointment to approximate the midpoint between clinic visits when the AYA may have decreased adherence; for 35 days total), resulting in *bursts* of EMA data collection [45]. Burst designs such as this have been used to study other AYA health behaviors over a longer period [46]. Each AYA will receive a MEMS TrackCap (AARDEX Group), a validated electronic adherence monitor that was also used in the Children’s Oncology Group study, to measure daily 6-MP adherence across the entire study period.

EMA surveys are brief (<1 minute to complete) and assess intrapersonal contexts (eg, individual-level variables, such as nausea, fatigue, and positive or negative affect) and interpersonal contexts (eg, family and social environment variables, such as recent family interaction and location). Enrolled AYAs will complete 11 daily survey questions, and their caregivers will complete four survey questions (see Table 1 for a complete list of survey items).

**Table 1:** Ecological momentary assessment (EMA) survey questions.

Variable			Informants		Items		Description
**Intrapersonal contexts**							
	Physical symptoms	AYA^a^		How much pain are you currently experiencing? How much fatigue are you currently experiencing? How much nausea are you currently experiencing?		Three items assessing the intensity of current pain, fatigue, and nausea on a scale of 0 (not at all) to 4 (extremely); adapted from two adult oncology EMA studies [47,48]	
	Positive and negative effect	AYA and caregiver		How positive were you feeling (happy or joyful) just before you received this text message? How negative were you feeling (stressed, mad or angry, nervous or anxious, or sad) were you feeling just before you received this text message?		Two items assessing the degree of positive affect and negative affect on a scale of 0 (not at all) to 4 (extremely); adapted from a physical activity EMA study [49,50]	
	Adherence motivation	AYA		How motivated are you to take 6-MP^b^ today?		One item assessing motivation to take 6-MP on a scale of 0 (not motivated) to 4 (extremely motivated); adapted from EMA study of medication adherence in adults with HIV [51]	
**Interpersonal and environmental contexts**							
	Family or social stressors	AYA and caregiver		In the past 24 hours, have you Had a misunderstanding or disagreement with your parent or child? How easy was it to talk to your parent or child about your thoughts and feelings? (AYA only) How lonely are you currently feeling?		Three adapted items from the Hassles Scale for Children [52] and an EMA study of adolescents with asthma [53], assessing whether or not the AYA experienced a disagreement or misunderstanding with parents and ease with communication and loneliness on a scale of 0 (not at all) to 4 (extremely)	
	Location or social company	AYA		Where were you right before you received this survey? Who were you with just before you received this survey?		Two items assessing where the AYA was (home, school, car, outdoors, restaurant, store, someone else’s house, gym, or someplace else) and who they were with (alone, mom or dad, sister or sisters, brother or brothers, other family, friend or friends, classmate or classmates, or someone else); adapted from EMA studies of diabetes adherence [54], physical activity [49,50], and asthma symptoms [53]	

^a^AYA: adolescent and young adult.

^b^6-MP: 6-mercaptopurine.

### Setting and Recruitment

We are currently recruiting eligible participants at the Children’s Hospital of Philadelphia (CHOP) Cancer Center during routine maintenance chemotherapy outpatient visits. Eligible participants will be provided with study advertising materials, including a study flier and a live demonstration of the ADAPTS app. Considering the COVID-19 pandemic, the protocol also includes procedures that allow for remote recruitment (eg, enrollment over the phone with verbal consent).

### Inclusion and Exclusion Criteria

This study will include 30 AYAs with leukemia or lymphoma in the maintenance phase of treatment and 30 of their matched caregivers. The inclusion criteria for AYAs are as follows: (1) aged 14-25 years; (2) diagnosed with acute lymphoblastic leukemia or lymphoma; (3) in the maintenance phase of treatment and completed at least 1 month of maintenance chemotherapy with at least 6 months remaining; (4) prescribed daily oral 6-MP; (5) English language proficiency; and (6) AYAs aged <18 years must have a caregiver (parent or legal guardian) to provide informed consent. The exclusion criterion for AYAs is cognitive impairments that would limit their ability to complete measures, as determined by their medical team. The inclusion criteria for caregivers are as follows: (1) nominated by an AYA as a caregiver involved in oncology care and (2) lives in the same household as the AYA (given this study’s focus on family-level processes that could affect 6-MP adherence).

### App Platform and Refinement Process

EMA surveys will be delivered via an open-source app called ADAPTS. ADAPTS is a modified version of an existing app called SARA [38]. In the original trial, SARA delivered daily surveys to AYA at risk for substance abuse for 30 days and provided a variety of theory-informed and developmentally relevant engagement strategies. Specifically, in addition to small amounts of money (US $1 for every 3 consecutive days of daily self-reporting), SARA provided nonmonetary incentives for completing surveys that were grounded in operant conditioning principles (eg, reinforcing survey completion with funny memes) and had a user-centered and gamified data collection environment (ie, an aquarium that became increasingly complex by unlocking fish as users completed surveys). To promote reciprocity, SARA also provided an unsolicited reward (an inspirational celebrity quote) before delivering the EMA survey. This app demonstrated slightly lower levels of EMA engagement compared with other substance abuse studies but with much smaller financial incentives (mean 62.3% surveys completed, mean 18.1 out of 30 days, SD 9.2; mean US $6.24, SD 3.83; range, US $1.00-$13.00 money earned). Engagement strategies were microrandomized to determine whether a particular strategy (eg, rewarding a meme after a survey was completed) proximally affected survey completion in the next time window (eg, the next day). The results provided preliminary support for the use of a reciprocity strategy in future trials.

Informed by user-centered design and agile science principles [40,55], our research team iteratively refined SARA for AYAs with cancer over four stages. First, we convened a multidisciplinary team of behavioral scientists and technologists to identify possible app modifications that could improve the app’s functionality and accommodate differences in this study’s methods or population. The following app modifications were made: (1) expanded the app to accommodate this study’s longer EMA period of 63 days (eg, including progressing the environment to grow from an aquarium into other levels); (2) developed a parallel caregiver version; (3) identified and vetted contemporary engagement content that would be particularly relevant to AYAs with cancer (ie, new memes and celebrity quotes); (4) delivered a survey reminder if EMA was not completed; and (5) incorporated one new engagement strategy—altruistic thank you messages for completing surveys (an other-benefitting incentive [37] that targets intrinsic motivations for helping other AYAs with cancer).

Second, stakeholders, including a convenience AYA sample (4 AYAs with a history of cancer involved in a hospital-based steering committee and 1 AYA without cancer) and 18 AYA oncology providers and research staff, rated 66 memes and 100 celebrity quotes identified from social media or web searches on a 5-point scale (from 1=“Do not like at all, do not recommend including” to 5=“Really like, use this”), which were narrowed to the most highly rated (>3.5/6) content (resulting in a final bank of 31 memes and 72 quotes). Third, we conducted a 1-month internal pilot test with oncology providers and research staff (n=8) to assess app glitches and elicit further feedback on the design and features. Research staff observed technical glitches (eg, surveys not registering when the device was offline and reminders sent at the wrong times) and provided additional suggestions for improving the app’s functionality (eg, storing previously received rewards in a feed like other popular social media apps). These glitches and suggestions were addressed before a 1-month pilot with AYAs with cancer (n=10). Finally, in the pilot with end users, AYAs generally reported high satisfaction with the app, and a few remaining technical problems were resolved (eg, malfunction with the survey submit button).

### Study App (ADAPTS)

**Figure 1:**
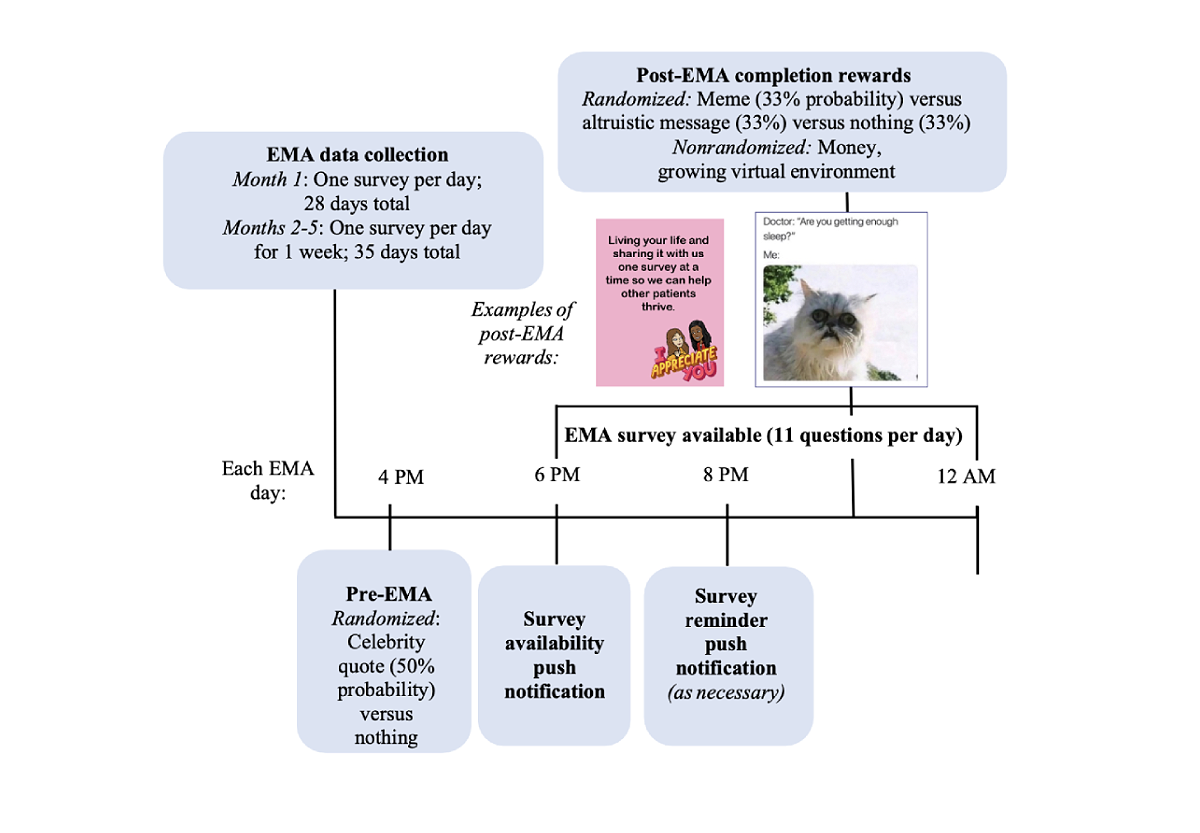
Schedule of ecological momentary assessment and engagement features for adolescent and young adult participants. EMA: ecological momentary assessment.

**Figure 2:**
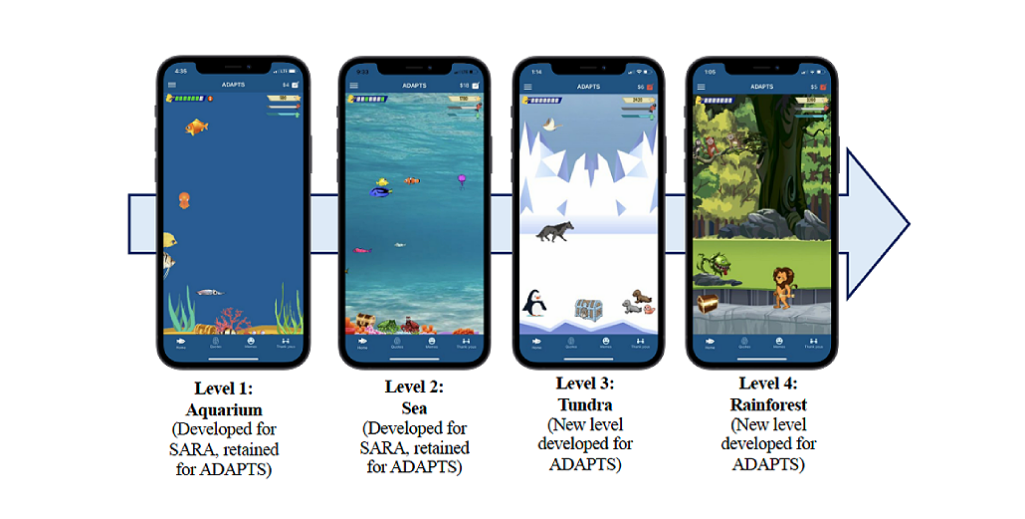
App environment expansion. ADAPTS: Adherence Assessments and Personalized Timely Support.

The finalized version of ADAPTS delivers EMA surveys to the AYAs and their caregivers, as well as several engagement strategies (Figure 1). The app is available for both iPhone and Android users. Surveys are available between 6 PM and midnight. If the surveys are not completed by 8 PM, participants will receive a reminder. Automated engagement strategies will include the following: (1) pre-EMA nonmonetary engagement strategies (inspirational celebrity quotes and survey reminders; Multimedia Appendix 1 for a complete list of quotes); (2) post-EMA nonmonetary rewards (funny memes, altruistic messages, and a growing virtual environment from an aquarium to an ocean, tundra, and then a rainforest; Figure 2 and Multimedia Appendices 2 and 3 for complete lists of memes and altruistic messages); and (3) post-EMA small monetary rewards (US $2 for completing surveys on every first day of a new EMA cycle, then US $1 for every 3-day streak with 100% completion of surveys).

**Figure 3:**
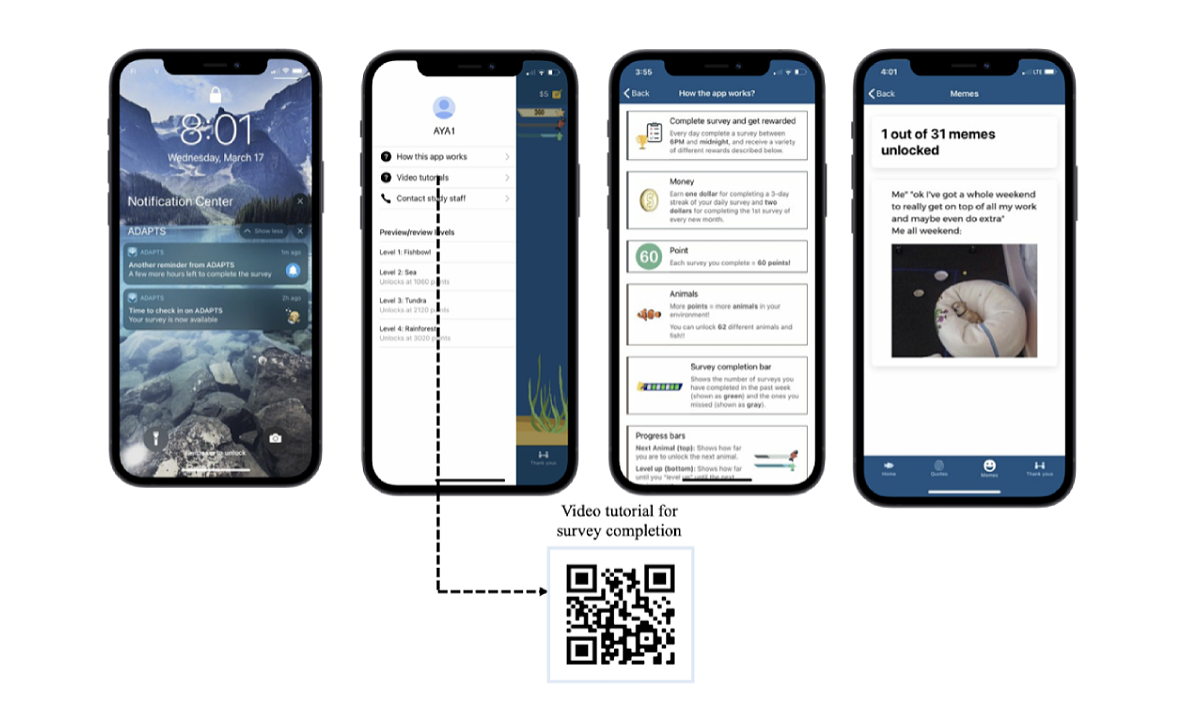
Other app features: push notifications, education, and reward storage.

Participants can provide feedback on certain engagement content (ie, celebrity quotes, memes, and altruistic messages) by liking or disliking each with a *thumbs up* or *thumbs down* button. Previously received engagement content will be stored in a feed. Each time participants unlock an animal or other content (eg, snow in the tundra) in the virtual environment (72 total, for approximately 1 new addition per EMA day), they will receive a fun fact (eg, “No penguins live in the north pole”; see Multimedia Appendix 4 for a complete list of animals and facts). Some content in the virtual environment is interactive, such as animals jumping when they are touched and turning on and off snow or rain. Other included app content includes a *how this app works* visual; video tutorials; a contact study staff page to directly call, text, or email the study coordinator; and survey completion and app progress bars to provide feedback and show when the participant will *level up* (Figure 3).

### Microrandomization

Before the EMA survey being sent, participants will be randomized every day at 4 PM, with a probability of 0.5, to receive a push notification with an inspirational celebrity quote or to receive nothing (Figure 1). Consistent with the original SARA app, this incentive will be sent before the EMA survey is available to facilitate reciprocity; that is, to increase the likelihood that participants will *return the favor* by completing the survey when it is later available. After the EMA survey is completed, participants will be randomized with a 0.33 probability of receiving a funny meme (targeting extrinsic motivation), an altruistic message (targeting intrinsic motivation), or nothing. The other engagement strategies (ie, money and growing virtual environments) will not be randomized. The caregiver version of the app includes nonmonetary rewards (same growing virtual environment) and small monetary rewards (same compensation schedule) but does not include AYA-focused memes or quotes.

### Human Support and Monitoring

In addition to automated engagement strategies integrated within the app, there are a few prompts that are manually sent by a member of our study team. First, after completing the first week of EMA, a standardized SMS text message will be sent to each AYA or caregiver participant to inquire about app glitches and provide a reminder to use the electronic pill bottle. Second, when a participant does not answer surveys for 3 consecutive days, they will receive an additional standardized SMS text message offering in-the-moment support for any app glitches for a maximum of one time during each EMA burst. Third, each month, a research assistant will meet briefly with families during monthly clinic visits to remind them of the study, ensure that the family is refilling MEMS bottles with 6-MP, confirm that AYAs and caregivers still have access to ADAPTS, provide financial compensation for their participation in the prior month, and download adherence data from the prior month as well as share with the AYA’s primary oncology provider. This level of human support is similar to our other prior mHealth trials [31], including the SARA trial.

To monitor for technical glitches, study staff will pull survey and engagement data each day to review whether (1) participants completed the survey from the prior day, (2) the money earned corresponds with the intended payment schedule, (3) the push notifications were sent and at the right times, and (4) participants are using the most up-to-date version of the app. Study staff will also review the list of active study participants and manually turn surveys *on* and *off* depending on their EMA burst cycle. Any technical issues will be documented in a log and shared with a technology support team within 24 hours.

### Data Management and Security

Raw EMA and app engagement data do not contain Protected Health Information and will be temporarily stored in an encrypted form, with a unique study ID, in the ADAPTS app on the smartphone. When connected to the internet, encrypted data will be transferred continuously to CHOP’s secure Enterprise Amazon Web Services S3 data repository, which supports data redundancy; local EMA data on the smartphone will be deleted after successful transfer. Data will be backed up each night to a separate S3 data repository. If a participant does not connect to the internet, then the data will be temporarily stored on their smartphone. The research team also developed a password-protected study monitoring website on the CHOP network. The study monitoring website is a tool for quickly reviewing each participant’s EMA completion rates (depicts survey completion at the daily and aggregate levels) and money earned across EMA days. It is also where the RA can turn surveys on or off.

### Primary Outcome

The primary outcome is daily 6-MP adherence, measured using a validated electronic medication bottle called MEMS TrackCap. MEMS TrackCaps have two parts: (1) a standard plastic vial that stores pills and (2) a lid that contains a sensor that registers times when the vial lid is opened and closed. This method of electronic adherence monitoring has been validated in pediatric cancer, has shown consistent accuracy in independent testing, and was the same measure used in the Children’s Oncology Group study that established a 95% critical adherence level needed for relapse prevention [1,56,57]. The accompanying medAmigo software (AARDEX Group) displays the timestamped adherence data for each day. Participants will be instructed to place 6-MP in the MEMS within 24 hours of enrollment, use the MEMS each day for the full duration of the 6-month study (rather than a pillbox or pharmacy bottle), and only open the bottle if they are taking 6-MP or refilling the bottle at that time. For each day, 6-MP adherence will be classified as 1 (took dose) or 0 (missed dose).

### Secondary (Exploratory) Outcome

We will explore whether the microrandomized engagement content proximally affects EMA survey completion, classified as 1 (completed survey) or 0 (did not complete survey). For the celebrity quotes delivered at 4 PM, the proximal outcome is whether the survey is completed in the evening on the same day. For the post-EMA rewards (altruistic thank you messages and memes), the proximal outcome is whether the survey is completed on the following day.

### Primary Data Analyses

This data set will consist of 30 AYA-caregiver dyads×63 days=a maximum of 1890 daily observations. EMA predictors include data from AYAs, caregivers, or dyadic data from both reports (eg, agreement between AYAs and caregivers that they had a disagreement that day). Tables and graphs will be created to demonstrate how 6-MP adherence varies by individual (physical symptoms, mood, and motivation), family (family disagreements and problems with communication), and social-environmental factors (time of day, day of the week, month of the year, where AYAs were, and who they were with). Mixed effects models will be used through SAS PROC GLIMMIX (SAS Institute) to examine whether EMA of contextual factors predicted the binary daily 6-MP adherence outcome. Separate mixed effects models will be constructed for different predictors and include the predictor as the fixed effect and a random intercept for each participant to account for between-person variability. Mixed effects models may also include demographic and treatment covariates (eg, race, ethnicity, and time since cancer diagnosis) as fixed effects if they demonstrated a significant association with the outcome. We will decompose the between-subject and within-subject effects of EMA of contextual factors by creating two predictors from the original score: (1) the individual mean across all time points (between-subject predictor) and (2) the deviation of the daily score from the individual mean (within-subject predictor). Given the embedded engagement strategies, we expect minimal missing data, and mixed effects modeling will be able to use all available outcome data and provide valid inferences if missingness is at random. However, we will examine the amount and pattern of missing data, and if missing is suspected to be not at random, sensitivity analyses will be performed to assess the robustness of the primary analysis results. Significant findings will inform tailoring variables for future JITAIs (eg, tailor messages based on affect, motivation, and day of week).

### Exploratory Analyses

We will analyze microrandomized trial data with a regression-based approach that was specifically developed to ensure unbiased estimates of the causal effects of time-varying treatments [58]. These analyses pool time-varying, longitudinal data across participants and use a log-link function to accommodate the binary outcome (survey completed vs not completed). The causal effect is expressed on the risk-ratio scale that measures the probability of proximally completing an EMA survey when an engagement strategy is deployed, divided by the probability of proximally completing an EMA survey when the engagement strategy was not deployed. To test the effect of reciprocity, proximal survey completion refers to the current day. For nonmonetary reinforcements that are delivered after the EMA survey is completed (memes and altruistic messages), proximal survey completion refers to the next day. Two separate analyses will be conducted: (1) examining the main effect of reciprocity (vs nothing) with the celebrity quote and (2) examining the main effects of the meme reinforcer versus altruistic message versus nothing. The risk ratio will be >1 if offering (vs not offering) the engagement strategy has a causal effect on the probability of proximal survey completion. These exploratory analyses will prescreen engagement components to retain in the JITAIs.

### Sample Size and Power

Power calculations were performed using the PASS 2021 [59]. With 63 days of repeated measures and assuming a moderate within-subject correlation of 0.5, the most effective sample size for predicting our binary adherence outcome (took dose vs not) is 59 AYAs [60]. However, our study sample size is constrained by the available AYAs who meet the inclusion criteria at our single institution. With our proposed sample size of 30 AYA-caregiver dyads, assuming a two-sided type 1 error of 0.05 and an approximate nonadherence rate of 20% (based on estimates from prior studies) [28,61], we will have 80% power to detect an odds ratio of 2.8 (a large effect) for every 1 SD increase in a continuous predictor [62].

## Results

This study received funding from the National Cancer Institute on September 1, 2019 (K08CA241335), institutional review board approval at CHOP on September 24, 2019, and began recruiting in June 2020. To date, of the proposed 30 AYA-caregiver dyads, 18 (60%) have been enrolled, and 15 (50%) have completed the 6-month study. We expect data collection to be completed by June 2022.

## Discussion

### Overview

In response to empirical and clinical data that have demonstrated suboptimal 6-MP adherence rates among AYAs and increased cancer relapse risk [1], we designed this app-based EMA study to identify the states that change rapidly (eg, mood and fatigue) and proximally affect an AYA’s adherence to taking the prescribed daily oral 6-MP. As a pervasive threat to mHealth is declining user engagement, we have incorporated theory-informed and user-centered engagement features within the app, and we will explore their proximal relationships with EMA engagement. Together, these aims represent important first steps toward translating daily contextual data into personalized and engaging adherence support. As such, our protocol is consistent with a paradigm shift toward precision health [19] and the first stages of the Behavior Change Theories, User-Centered Design, and Social Marketing and multiphase optimization strategy intervention development frameworks [40,41]. Study innovations also include (1) collecting multilevel EMA data from both AYAs and their caregivers, which is informed by a social-ecological theory of disease self-management; (2) obtaining EMA data in bursts over a longer period, with support from low-cost engagement strategies; (3) using an open-source EMA app, which is cost effective and increases the generalizability of methods; and (4) microrandomizing EMA engagement strategies to explore their proximal impact on survey completion, helping lay the groundwork for selecting engagement components for the later JITAIs.

An optimized JITAI is well suited to address the gaps in prior adherence-promotion interventions by maximizing engagement, minimizing burden, and delivering a personalized intervention only when there is a true benefit [35]. Consistent with the broader pediatric adherence-promotion literature [42,43], effect sizes from a few existing and static 6-MP adherence-promotion interventions are small and possess key limitations (eg, suboptimal participant engagement in digital health components and relying on parental supervision of medication taking, which may not be feasible or acceptable to an AYA population) [63,64]. Moreover, optimization questions regarding which intervention option to offer to AYAs and when have been neglected. The results from this EMA study will serve as an initial check of the time-varying contexts that are salient for 6-MP adherence, which will help inform the EMA questions that will be retained and used as tailoring variables in the JITAIs. Moreover, this study will help prescreen engagement components that will be further piloted in the intervention.

### Future Directions

The next phase of this 6-MP adherence research is to engage with a multidisciplinary research team (including experts in behavioral science, mHealth, oncology, AYA development, and health communications), along with AYA cancer representatives, to identify an initial set of tailoring variables, decision rules, and self-management intervention options for the JITAIs. For example, a tailoring variable may be *adherence motivation*, a self-management intervention component may be *goal congruence*, and a decision rule may be *deliver if motivation decreases by X points*. We will use an intervention mapping–informed approach [65,66] to ensure that each JITAI component is grounded in self-management theory and existing empirical evidence (including results from the present EMA study). Mobile messages that map onto the tailoring variables, decision rules, and intervention components will then be created through an iterative process with the research team and AYA stakeholders, with members suggesting and revising the content to be theory grounded, developmentally appropriate, specific to intervention goals, and at a sixth grade reading level. A smaller subset of AYAs from the EMA study will be invited to participate in a focus group to provide feedback on this initial prototype.

Another future direction is understanding the contexts (eg, mood, family interactions, and weekends) in which mHealth engagement strategies are the most effective. Data from this study and others could inform reinforcement learning algorithms to increase the probability of providing an engagement strategy that is the most effective for an individual in a particular context. It will also be important to build an evidence base to understand the generalizability of engagement strategies across populations. For example, it is possible that our population—AYAs who have recently entered remission for their cancer treatment—may be more responsive to altruistic reinforcements that remind them that they are helping other cancer patients, compared with other AYA groups. To achieve effective adherence to JITAIs, we also need to develop effective engagement JITAIs to promote the uptake of adherence-promoting content.

### Limitations and Anticipated Challenges

First, although our study focuses on adherence to the most common oral chemotherapy prescribed to pediatric cancer patients (6-MP), it is not designed to assess adherence to other cancer-related medications (eg, prophylactic antibiotics). AYAs taking 6-MP as maintenance chemotherapy are an exemplar relatively homogenous disease cohort with known adherence challenges with which to test new methods of adherence observation and intervention. However, expansion to other AYA oncology populations, who have understudied adherence patterns, will be essential in future studies. Second, our sample size and power are not optimal to detect small to medium effects. Although our cancer center is one of the largest pediatric centers in North America, the age and diagnosis inclusion criteria for our study are strict (to reduce heterogeneity), which narrows the available AYA population to recruit. Moreover, our observation period of 63 days does not capture the full duration of maintenance chemotherapy in this population (approximately 18 months). Nonetheless, this study nearly doubles the number of participants and daily observations that were included in our pilot EMA study [28] and offers an important opportunity to replicate significant findings. It will be important that a later JITAI optimization trial include multiple sites to recruit a larger and more demographically diverse sample of AYAs who are prescribed 6-MP. Third, engagement content can come in and out of vogue very quickly for an AYA population (eg, the shelf life of popular memes may be short, and high circulation of popular memes may reduce novelty). We vetted and selected content that AYAs liked and perceived as rewarding; however, it is possible that they will become less desirable over time. Fourth, additional tailoring of engagement strategies is likely needed because of individual preferences. Finally, there may be feasibility challenges with an app-based intervention that is dependent on the completion of EMA surveys.

### Conclusions

Together, this study will determine the temporal associations between daily contextual factors on 6-MP adherence and explore the proximal impact of various engagement strategies on EMA survey completion. The execution of this protocol represents an important first step toward translating daily, contextual data into personalized and engaging adherence support. Our future JITAI will combine adaptive strategies to promote treatment adherence while concurrently adapting engagement strategies for promoting EMA completion, which may be a generalizable blueprint for improving the impact and reach of other AYA mHealth interventions.

## Appendix

Multimedia Appendix 1 Celebrity quotes.

Multimedia Appendix 2 Memes.

Multimedia Appendix 3 Altruistic messages.

Multimedia Appendix 4 Animal facts.

Multimedia Appendix 5 Peer-reviewer report from National Cancer Institute NCI - Subcommittee J - Career Development (National Institutes of Health).

## Abbreviations

6-MP: 6-mercaptopurine
ADAPTS: Adherence Assessments and Personalized Timely Support
AYA: adolescent and young adult
CHOP: Children’s Hospital of Philadelphia
EMA: ecological momentary assessment
JITAI: just-in-time adaptive intervention
mHealth: mobile health
REDCap: Research Electronic Data Capture

## References

[1] Bhatia S, Landier W, Hageman L, Kim H, Chen Y, Crews KR, Evans WE, Bostrom B, Casillas J, Dickens DS, Maloney KW, Neglia JP, Ravindranath Y, Ritchey AK, Wong FL, Relling MV (2014). 6MP adherence in a multiracial cohort of children with acute lymphoblastic leukemia: a Children's Oncology Group study. Blood.

[2] Gupta S, Bhatia S (2017). Optimizing medication adherence in children with cancer. Curr Opin Pediatr.

[3] Butow P, Palmer S, Pai A, Goodenough B, Luckett T, King M (2010). Review of adherence-related issues in adolescents and young adults with cancer. J Clin Oncol.

[4] Smith AW, Seibel NL, Lewis DR, Albritton KH, Blair DF, Blanke CD, Bleyer WA, Freyer DR, Geiger AM, Hayes-Lattin B, Tricoli JV, Wagner LI, Zebrack BJ (2016). Next steps for adolescent and young adult oncology workshop: An update on progress and recommendations for the future. Cancer.

[5] (2006). Closing the gap: Research and care imperatives for adolescents and young adults with cancer. National Cancer Institute and LIVESTRONG Young Adult Alliance.

[6] Wu YP, Stenehjem DD, Linder LA, Yu B, Parsons BG, Mooney R, Fluchel MN (2018). Adherence to oral medications during maintenance therapy among children and adolescents with acute lymphoblastic leukemia: a medication refill analysis. J Pediatr Oncol Nurs.

[7] Arnett JJ (2000). Emerging adulthood: A theory of development from the late teens through the twenties. Am Psychol.

[8] Coccia PF, Pappo AS, Beaupin L, Borges VF, Borinstein SC, Chugh R, Dinner S, Folbrecht J, Frazier AL, Goldsby R, Gubin A, Hayashi R, Huang MS, Link MP, Livingston JA, Matloub Y, Millard F, Oeffinger KC, Puccetti D, Reed D, Robinson S, Rosenberg AR, Sanft T, Spraker-Perlman HL, von Mehren M, Wechsler DS, Whelan KF, Yeager N, Gurski LA, Shead DA (2018). Adolescent and Young Adult Oncology, Version 2.2018, NCCN Clinical Practice Guidelines in Oncology. J Natl Compr Canc Netw.

[9] Steinberg L (2016). Risk taking in adolescence. Curr Dir Psychol Sci.

[10] Morrison CF, Pai AL, Martsolf D (2018). Facilitators and barriers to self-management for adolescents and young adults following a hematopoietic stem cell transplant [Formula: see text]. J Pediatr Oncol Nurs.

[11] Landier W, Hughes CB, Calvillo ER, Anderson NL, Briseño-Toomey D, Dominguez L, Martinez AM, Hanby C, Bhatia S (2011). A grounded theory of the process of adherence to oral chemotherapy in Hispanic and caucasian children and adolescents with acute lymphoblastic leukemia. J Pediatr Oncol Nurs.

[12] Kennard BD, Stewart SM, Olvera R, Bawdon RE, hAilin AO, Lewis CP, Winick NJ (2004). Nonadherence in adolescent oncology patients: Preliminary data on psychological risk factors and relationships to outcome. J Clin Psychol Med Sett.

[13] McGrady M, Prosser LA, Thompson AN, Pai AL (2018). Application of a discrete choice experiment to assess adherence-related motivation among adolescents and young adults with cancer. J Pediatr Psychol.

[14] McGrady ME, Brown GA, Pai AL (2016). Medication adherence decision-making among adolescents and young adults with cancer. Eur J Oncol Nurs.

[15] Hullmann SE, Brumley LD, Schwartz LA (2015). Medical and psychosocial associates of nonadherence in adolescents with cancer. J Pediatr Oncol Nurs.

[16] Pritchard M, Butow PN, Stevens MM, Duley JA (2006). Understanding medication adherence in pediatric acute lymphoblastic leukemia: a review. J Pediatr Hematol Oncol.

[17] Psihogios AM, Schwartz LA, Ewing KB, Czerniecki B, Kersun LS, Pai AL, Deatrick JA, Barakat LP (2020). Adherence to multiple treatment recommendations in adolescents and young adults with cancer: a mixed methods, multi-informant investigation. J Adolesc Young Adult Oncol.

[18] Partridge A, Avorn J, Wang PS, Winer EP (2002). Adherence to therapy with oral antineoplastic agents. J Natl Cancer Inst.

[19] Wongvibulsin S, Martin SS, Saria S, Zeger SL, Murphy SA (2020). An individualized, data-driven digital approach for precision behavior change. Am J Lifestyle Med.

[20] Spring B, Gotsis M, Paiva A, Spruijt-Metz D (2013). Healthy apps: Mobile devices for continuous monitoring and intervention. IEEE Pulse.

[21] Rivera DE, Jimison HB (2013). Systems modeling of behavior change: Two illustrations from optimized interventions for improved health outcomes. IEEE Pulse.

[22] Saranummi N, Spruijt-Metz D, Intille SS, Korhonen I, Nilsen WJ, Pavel M (2013). Moving the science of behavioral change into the 21st century: Part 2. IEEE Pulse.

[23] Spruijt-Metz D, Hekler E, Saranummi N, Intille S, Korhonen I, Nilsen W, Rivera DE, Spring B, Michie S, Asch DA, Sanna A, Salcedo VT, Kukakfa R, Pavel M (2015). Building new computational models to support health behavior change and maintenance: new opportunities in behavioral research. Transl Behav Med.

[24] Smyth JM, Stone AA (2003). Ecological momentary assessment research in behavioral medicine. J Happin Stud.

[25] Heron K, Everhart RS, McHale SM, Smyth JM (2017). Using mobile-technology-based Ecological Momentary Assessment (EMA) methods with youth: A systematic review and recommendations. J Pediatr Psychol.

[26] Smyth J, Heron KE, McHale M, Amato P, Booth A (2014). Ecological momentary assessment (EMA) in family research. Emerging Methods in Family Research.

[27] Bolger N, Laurenceau JP (2013). Intensive Longitudinal Methods: An Introduction to Diary and Experience Sampling Research.

[28] Psihogios AM, Li Y, Ahmed A, Huang J, Kersun LS, Schwartz LA, Barakat LP (2021). Daily text message assessments of 6-mercaptopurine adherence and its proximal contexts in adolescents and young adults with leukemia: A pilot study. Pediatr Blood Cancer.

[29] Li S, Psihogios AM, McKelvey ER, Ahmed A, Rabbi M, Murphy S (2020). Microrandomized trials for promoting engagement in mobile health data collection: Adolescent/young adult oral chemotherapy adherence as an example. Curr Opin Syst Biol.

[30] Wen CK, Schneider S, Stone AA, Spruijt-Metz D (2017). Compliance with mobile ecological momentary assessment protocols in children and adolescents: a systematic review and meta-analysis. J Med Internet Res.

[31] Psihogios A, King-Dowling S, O'Hagan B, Darabos K, Maurer L, Young J, Fleisher L, Barakat LP, Szalda D, Hill-Kayser CE, Schwartz LA (2021). Contextual predictors of engagement in a tailored mhealth intervention for adolescent and young adult cancer survivors. Ann Behav Med.

[32] Hightow-Weidman LB, Bauermeister JA (2020). Engagement in mHealth behavioral interventions for HIV prevention and care: making sense of the metrics. Mhealth.

[33] Mulvaney SA, Vaala S, Hood KK, Lybarger C, Carroll R, Williams L, Schmidt DC, Johnson K, Dietrich MS, Laffel L (2018). Mobile momentary assessment and biobehavioral feedback for adolescents with type 1 diabetes: feasibility and engagement patterns. Diabetes Technol Ther.

[34] Giles EL, Robalino S, McColl E, Sniehotta FF, Adams J (2014). The effectiveness of financial incentives for health behaviour change: systematic review and meta-analysis. PLoS One.

[35] Nahum-Shani I, Smith SN, Spring BJ, Collins LM, Witkiewitz K, Tewari A, Murphy SA (2018). Just-in-Time Adaptive Interventions (JITAIs) in mobile health: key components and design principles for ongoing health behavior support. Ann Behav Med.

[36] Wagner IB, Liu E, Shaw SD, Iakovlev GC, Zhou LM, Harrington C, Abowd G, Yoon C, Kumar S, Murphy S, Spring B, Nahum-Shani I (2017). wrapper: Operationalizing engagement strategies in mHealth. Proceedings of the 2017 ACM International Joint Conference on Pervasive and Ubiquitous Computing and Proceedings of the 2017 ACM International Symposium on Wearable Computers.

[37] Huang G (2020). Does warm glow promote physical activity? Examining the relative effectiveness of self-benefiting versus other-benefiting incentives in motivating fitness app use by corporate sponsorship programs. Health Commun.

[38] Rabbi M, Philyaw-Kotov M, Lee JR, Mansour A, Dent L, Wang X, Cunningham R, Bonar E, Nahum-Shani I, Klasnja P, Walton M, Murphy S (2017). Sara: a mobile app to engage users in health data collection. Proceedings of the 2017 ACM International Joint Conference on Pervasive and Ubiquitous Computing and Proceedings of the 2017 ACM International Symposium on Wearable Computers.

[39] Modi AC, Pai AL, Hommel KA, Hood KK, Cortina S, Hilliard ME, Guilfoyle SM, Gray WN, Drotar D (2012). Pediatric self-management: a framework for research, practice, and policy. Pediatrics.

[40] Patel S, Arya M (2017). The BUS Framework: A comprehensive tool in creating an mhealth app utilizing behavior change theories, user-centered design, and social marketing. J Mob Technol Med.

[41] Collins LM (2018). Optimization of Behavioral, Biobehavioral, and Biomedical Interventions: The Multiphase Optimization Strategy (MOST).

[42] Pai A, McGrady M (2014). Systematic review and meta-analysis of psychological interventions to promote treatment adherence in children, adolescents, and young adults with chronic illness. J Pediatr Psychol.

[43] Kahana S, Drotar D, Frazier T (2008). Meta-analysis of psychological interventions to promote adherence to treatment in pediatric chronic health conditions. J Pediatr Psychol.

[44] Kazak A, Alderfer M, Enlow PT, Lewis AM, Vega G, Barakat L, Kassam-Adams N, Pai A, Canter KS, Hildenbrand AK, McDonnell GA, Price J, Schultz C, Sood E, Phan TL (2021). COVID-19 exposure and family impact scales: factor structure and initial psychometrics. J Pediatr Psychol.

[45] Bhatia S, Landier W, Shangguan M, Hageman L, Schaible AN, Carter AR, Hanby CL, Leisenring W, Yasui Y, Kornegay NM, Mascarenhas L, Ritchey AK, Casillas JN, Dickens DS, Meza J, Carroll WL, Relling MV, Wong FL (2012). Nonadherence to oral mercaptopurine and risk of relapse in Hispanic and non-Hispanic white children with acute lymphoblastic leukemia: a report from the children's oncology group. J Clin Oncol.

[46] Patrick ME, Maggs JL, Lefkowitz ES (2015). Daily associations between drinking and sex among college students: A longitudinal measurement burst design. J Res Adolesc.

[47] Hacker ED, Ferrans CE (2007). Ecological momentary assessment of fatigue in patients receiving intensive cancer therapy. J Pain Symptom Manage.

[48] Ratcliff CG, Lam CY, Arun B, Valero V, Cohen L (2014). Ecological momentary assessment of sleep, symptoms, and mood during chemotherapy for breast cancer. Psychooncology.

[49] Dunton GF, Huh J, Leventhal AM, Riggs N, Hedeker D, Spruijt-Metz D, Pentz MA (2014). Momentary assessment of affect, physical feeling states, and physical activity in children. Health Psychol.

[50] Dunton G, Liao Y, Intille S, Wolch J, Pentz MA (2011). Physical and social contextual influences on children's leisure-time physical activity: an ecological momentary assessment study. J Phys Act Health.

[51] Cook P, Schmiege SJ, Starr W, Carrington JM, Bradley-Springer L (2017). Prospective state and trait predictors of daily medication adherence behavior in HIV. Nurs Res.

[52] Parfenoff S, Jose PE (1989). Measuring daily stress in children. ERIC Institute of Education Sciences.

[53] Dunton G, Dzubur E, Li M, Huh J, Intille S, McConnell R (2016). Momentary assessment of psychosocial stressors, context, and asthma symptoms in hispanic adolescents. Behav Modif.

[54] Borus JS, Blood E, Volkening LK, Laffel L, Shrier LA (2013). Momentary assessment of social context and glucose monitoring adherence in adolescents with type 1 diabetes. J Adolesc Health.

[55] Hekler EB, Klasnja P, Riley WT, Buman MP, Huberty J, Rivera DE, Martin CA (2016). Agile science: creating useful products for behavior change in the real world. Transl Behav Med.

[56] McGrady ME, Holbein CE, Smith AW, Morrison CF, Hommel KA, Modi AC, Pai AL, Ramsey RR (2018). An independent evaluation of the accuracy and usability of electronic adherence monitoring devices. Ann Intern Med.

[57] Rohan J, Fukuda T, Alderfer MA, Donewar CW, Ewing LW, Katz ER, Muriel AC, Vinks AA, Drotar D (2017). Measuring medication adherence in pediatric cancer: an approach to validation. J Pediatr Psychol.

[58] Qian T, Yoo H, Klasnja P, Almirall D, Murphy SA (2021). Rejoinder: 'Estimating time-varying causal excursion effects in mobile health with binary outcomes'. Biometrika.

[59] PASS 2021 Power analysis and sample size software. NCSS Statistical Software.

[60] Neuhaus JM, Segal MR (1993). Design effects for binary regression models fitted to dependent data. Stat Med.

[61] Pai AL, McGrady ME, Szulczewski L, Modi AC, Driscoll KA (2020). Pediatric oncology. Adherence and Self-Management in Pediatric Populations.

[62] Novikov I, Fund N, Freedman LS (2010). A modified approach to estimating sample size for simple logistic regression with one continuous covariate. Stat Med.

[63] Bhatia S, Hageman L, Chen Y, Wong FL, Mascarenhas L, Freyer DR, Mba N, Aristizabal P, Walterhouse D, Lew G, Kempert P, Russell T, McNall-Knapp R, Jacobs SS, Dang H, Raetz EA, Relling MV, Landier W (2019). A randomized trial of a mercaptopurine (6MP) adherence-enhancing intervention in children with acute lymphoblastic leukemia (ALL): A COG ACCL1033 study. J Clin Oncol.

[64] Kato PM, Cole SW, Bradlyn AS, Pollock BH (2008). A video game improves behavioral outcomes in adolescents and young adults with cancer: a randomized trial. Pediatrics.

[65] Bartholomew LK, Parcel GS, Kok G (1998). Intervention mapping: a process for developing theory- and evidence-based health education programs. Health Educ Behav.

[66] Cane J, Richardson M, Johnston M, Ladha R, Michie S (2015). From lists of behaviour change techniques (BCTs) to structured hierarchies: comparison of two methods of developing a hierarchy of BCTs. Br J Health Psychol.

