# Supplementary material for: Understanding Adolescent and Young Adult 6-Mercaptopurine Adherence and mHealth Engagement During Cancer Treatment: Protocol for Ecological Momentary Assessment
Source: JMIR Res Protoc. 2021 Oct 22;10(10):e32789. doi: 10.2196/32789 (PMC8571686; doi:10.2196/32789)
Supplement: Multimedia Appendix 1 [file resprot_v10i10e32789_app1.docx]

**Multimedia Appendix A: Celebrity Quotes** *(72 total)*

| **Celebrity** | **Quote** |
| --- | --- |
| Ali Krieger | I have fallen a few times, and each time, I have gotten up and grown as a person and looked at things a little differently. |
| Allen Iverson | You hit road blocks in life, but I'm proof that you can overcome those road blocks and become what you want to become. |
| Ariana Grande | Every time you're faced with something ugly, focus on something beautiful. What you focus on expands. Only you can change your reality. |
| Audrey Hepburn | The best thing to hold onto in life is each other. |
| Beyonce | Your self-worth is determined by you. You don't have to depend on someone telling you who you are. |
| Beyonce | It's not about perfection. It's about purpose. |
| Bo Burnham | Laughter is the best medicine beside, y'know, medicine. |
| Brie Larson | Maybe you're not perfect, but you're willing to actually look at yourself and take some kind of accountability. That's a change. It might not mean that you can turn everything around, but I think there's something incredibly hopeful coming your way. |
| Cardi B | Knock me down nine times, but I get up 10. |
| Casey Neistat | The most dangerous thing you can do in life is play it safe. |
| Chance the Rapper | Something I try to instill in others is to just be a good person. It's a decision you make a million times a day. But if you just keep trying, good stuff comes to you in an ordained way. |
| Chris Hemsworth | For me, life is about experience and being a good person. |
| Christian Dior | I think that we have to be aware that people are allowed to make mistakes in their life. |
| Conor McGregor | What defines us is how well we rise after falling. |
| Daisy Ridley | It's one thing for other people to see potential in you, and it's quite another for you to understand that and see it in yourself. |
| Danica Patrick | Sometimes, it takes looking at the past to really be able to move forward and learn from it. |
| David Beckham | The only time you run out of chances is when you stop taking them. |
| Demi Lovato | You're stronger than you think you are. Keep pushing forward, never give up on your dreams, and don't let anyone stop you. |
| Derek Jeter | Surround yourself with good people. People who are going to be honest with you and look out for your best interests. |
| Drake | Sometimes it's the journey that teaches you a lot about your destination. |
| Dumbledore | It is our choices, Harry, that show what we truly are, far more than our abilities. |
| Ellen DeGeneres | Be open to learning new lessons, even if they contradict the lessons you learned yesterday. |
| Emma Watson | Don't let anyone tell you what you can and can't do or achieve. Do what you want to do and be who you want to be. |
| Gigi Hadid | You are a shining star. Don't allow anyone to dim your brightness. |
| Greta Thunberg | We showed that we are united and that we young people are unstoppable. It is the only way. |
| J.R.R. Tolkien | The world is indeed full of peril, and in it there are many dark places; but still there is much that is fair, and though in all lands love is now mingled with grief, it grows perhaps the greater. |
| Jay-Z | You learn more in failure than you ever do in success. |
| Jimmy Rollins | Talent only goes so far. You have to work hard the rest of the way. |
| Joel Embiid | Trust the process. |
| Jonathan Van Ness | When you're willing to be vulnerable, you can surprise yourself at how strong you can be. |
| J.W. Stephens | Be the person your dog thinks you are. |
| Kawhi Leonard | I like being the underdog so they donÕt expect whatÕs going to happen. It pushes me to work harder and to do the things IÕm not doing better. |
| Kevin Hart | The only time you should look back in life is to see how far you have come. |
| Khalid | Positivity, confidence, and persistence are key in life, so never give up on yourself. |
| Lady Gaga | Don't you ever let a soul in the world tell you that you can't be exactly who you are. |
| LeBron James | Greatness is defined by how much you want to put into what you do. |
| Lili Rheinhart | You are the one thing in this world, above all other things, that you must never give up on. |
| Lorde | Like wildflowers, you must allow yourself to grow in the ways people never thought you would. |
| Malala Yousafzai | We were scared, but our fear was not as strong as our courage. |
| Martin Luther King Jr. | We must accept finite disappointment, but never lose infinite hope. |
| Maya Angelou | We may encouter many defeats but we must not be defeated. |
| Megan Rapinoe | No matter what life throws at you or how unfair you think it is, never give up. Pick yourself up and go on. |
| Meghan Markle | Reflecting on where I came from helps me to appreciate and balance what I have now. |
| Michael Jordan | My attitude is that if you push me towards something that you think is a weakness, then I will turn that perceived weakness into a strength. |
| Michael Phelps | Perseverance, determination, commitment, and courage Ð those things are real. The desire for redemption drives you. |
| Michelle Obama | The only limit to the height of your achievements is the reach of your dreams and your willingness to work hard for them. |
| Miley Cyrus | Let your light shine. Be a source of strength and courage. Share your wisdom. Radiate. |
| Neymar | The secret is to believe in your dreams; in your potential that you can be like your star, keep searching, keep believing and don't lose faith in yourself. |
| Nick Jonas | Life happens. Adapt. Embrace change, and make the most of everything that comes your way. |
| Nick Jonas | Live your life knowing that your potential is so much more than you can comprehend yourself. |
| Nicki Minaj | Your victory is right around the corner. Never give up. |
| Odell Beckham Jr. | Take advantage of every opportunity life gives you. |
| Post Malone | Surround yourself around people who want to see you grow. |
| Priyanka Chopra Jonas | Work hard, stay focused, and be committed. Most often than not, you'll come out on top. |
| Rihanna | It's nice to look back on your life and see things as lessons, and not regrets. |
| Rihanna | I'm gonna look back on my life and say that I lived it for me. |
| Robert Downey Jr. | Remember that just because you hit bottom doesn't mean you have to stay there. |
| Robin Williams | Make your life spectacular. |
| Ronda Rousey | To be the best, you have to constantly be challenging yourself, raising the bar, pushing the limits of what you can do. Don't stand still, leap forward. |
| Russell Wilson | Every setback has a major comeback. |
| Ryan Gosling | I've learned that it's important not to limit yourself. You can do whatever you really love to do, no matter what it is. |
| Ryan Howard | Sometimes, you're going to have to work hard, sometimes extra hard, and sometimes you still won't get that recognition. That's life. That's the way it is. But if you keep working, eventually you'll get there. |
| Shaq | Excellence is a habit. You are what you repeatedly do. |
| Shawn Mendes | My advice to anyone with a dream is to never stop pursuing it. |
| Simone Biles | We can push ourselves further. We always have more to give. |
| Tom Brady | I didn't come this far to only come this far, so we've still got further to go. |
| Tom Hardy | You mustn't be afraid to dream a little bigger, kid. |
| Tyrion Lannister | Never forget what you are. The rest of the world will not. Wear it like armor and it can never be used to hurt you. |
| Vera Wang | In the end, it’s not about failure, it’s about how much you love what you do. |
| Will Smith | The first step is you have to say that you can. |
| Zach Ertz | Stay focused, believe that you can achieve at the highest level, surround yourself with others who believe in you, and do not stray from your goal. |
| Zayn Malik | No matter how many times people try to criticize you, the best revenge is to prove them wrong. |
