# Supplementary figures and images for: Understanding Adolescent and Young Adult 6-Mercaptopurine Adherence and mHealth Engagement During Cancer Treatment: Protocol for Ecological Momentary Assessment
Source: JMIR Res Protoc. 2021 Oct 22;10(10):e32789. doi: 10.2196/32789 (PMC8571686; doi:10.2196/32789)

**Multimedia Appendix B: Memes** *(31 total)*

| 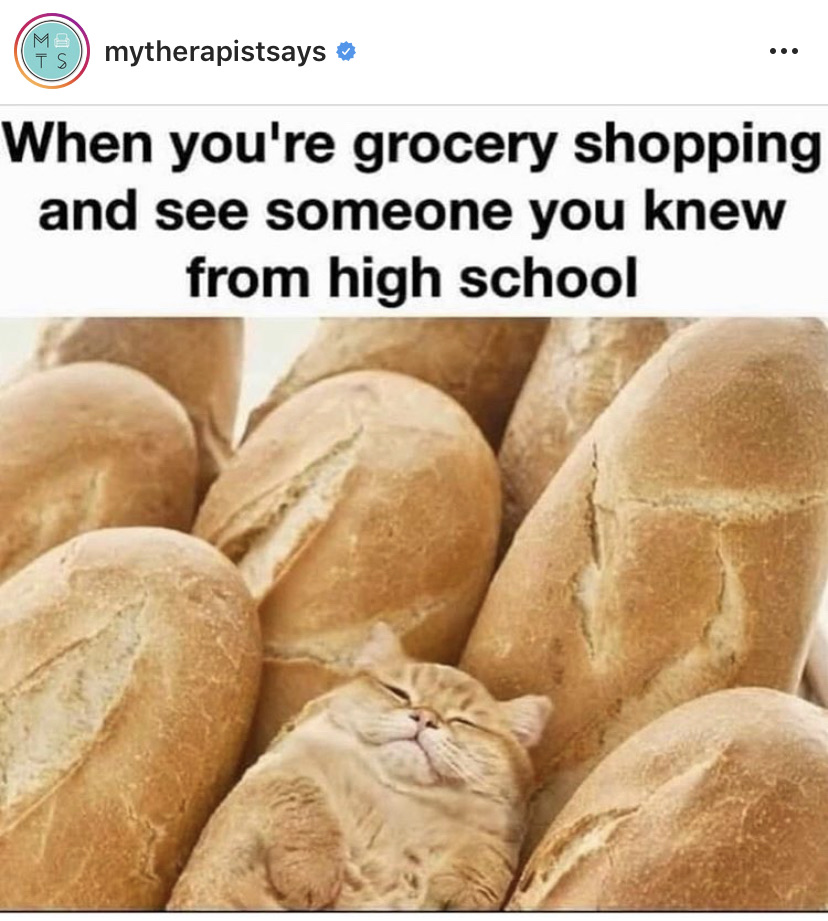 |
| --- |
| 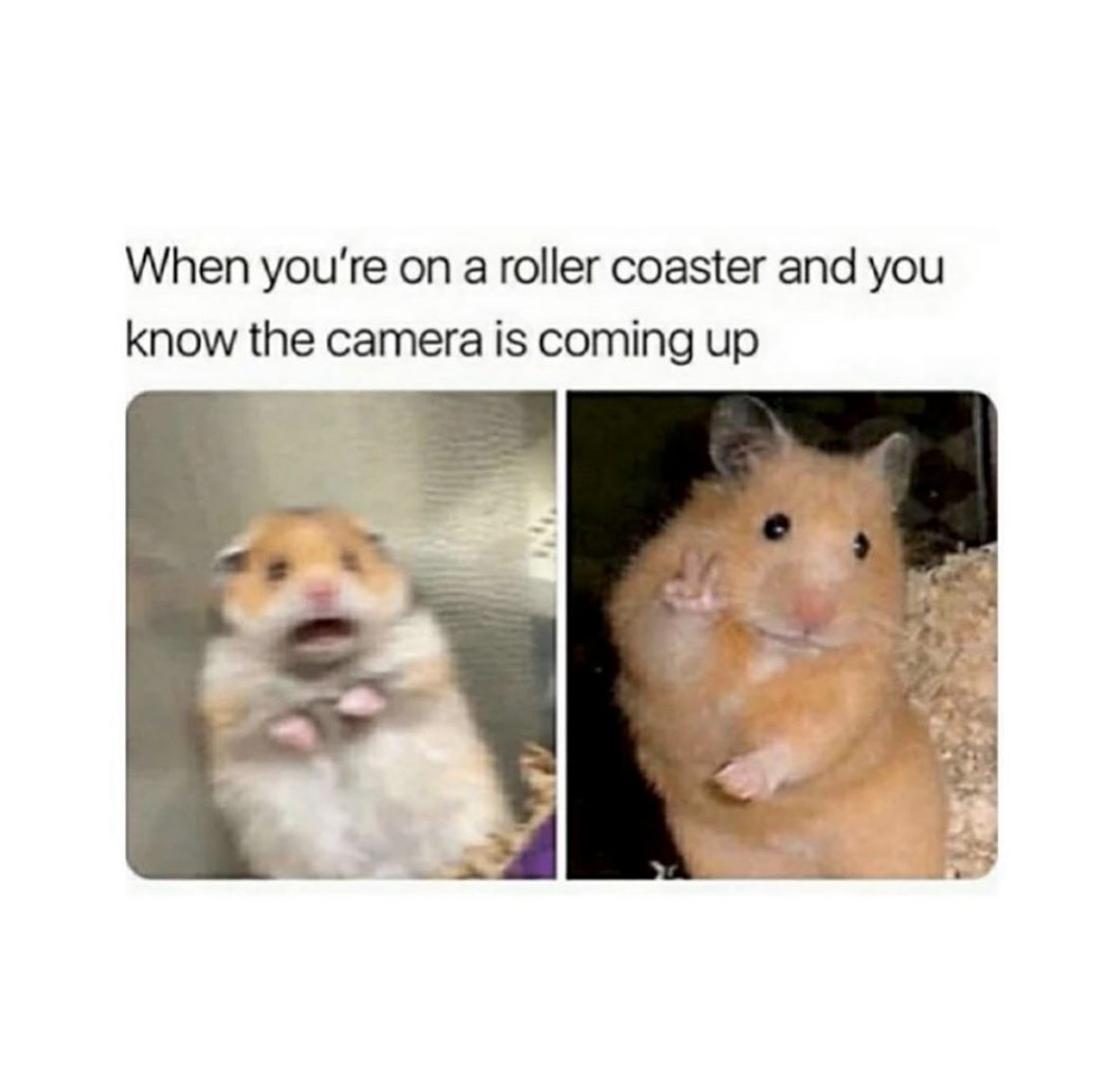 |
| 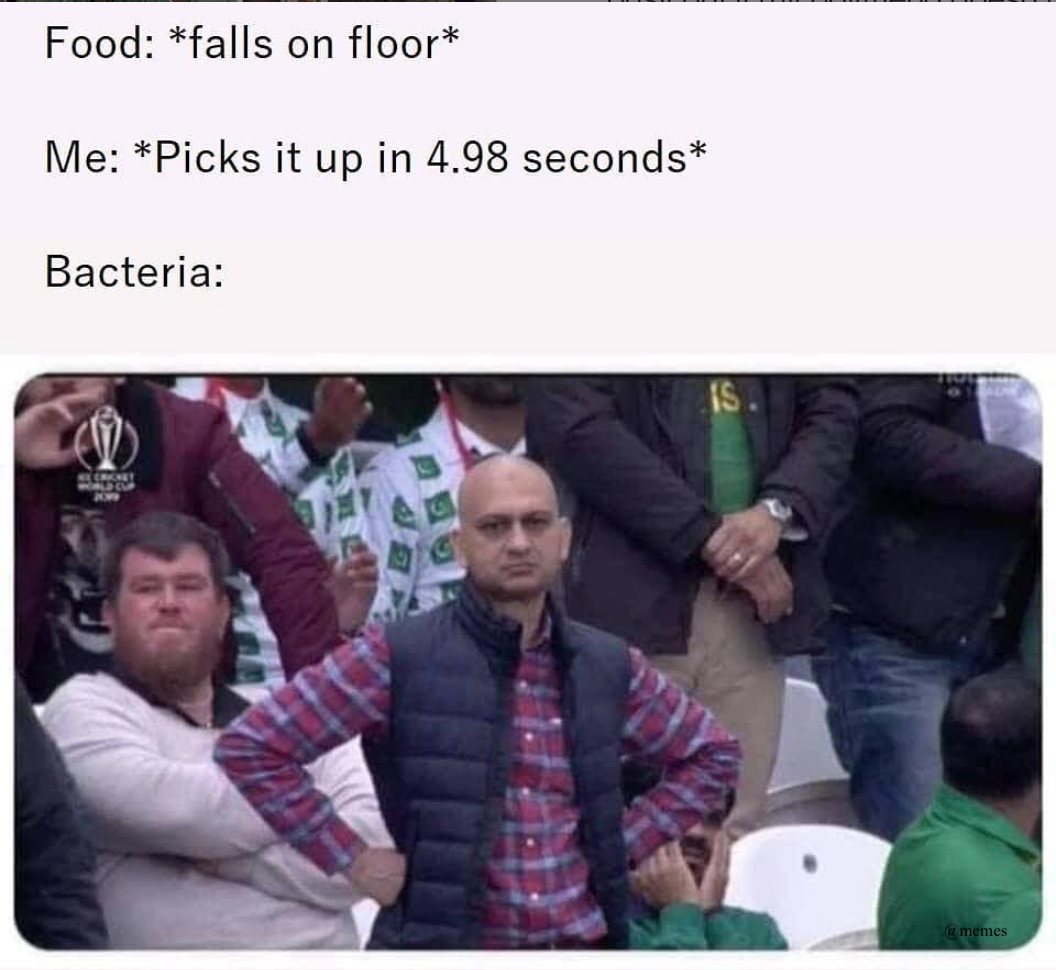 |
| 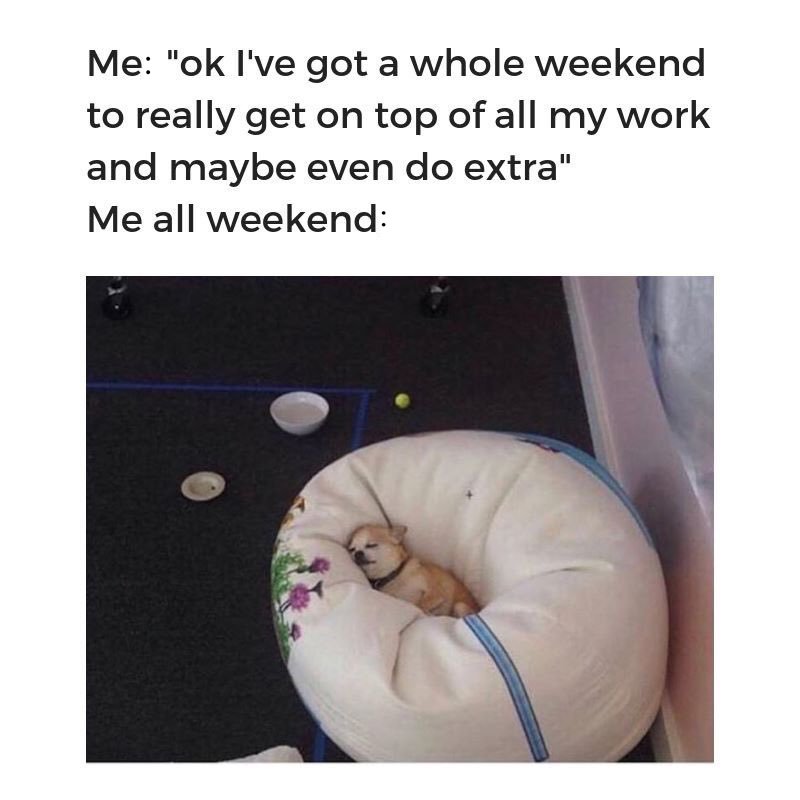 |
| 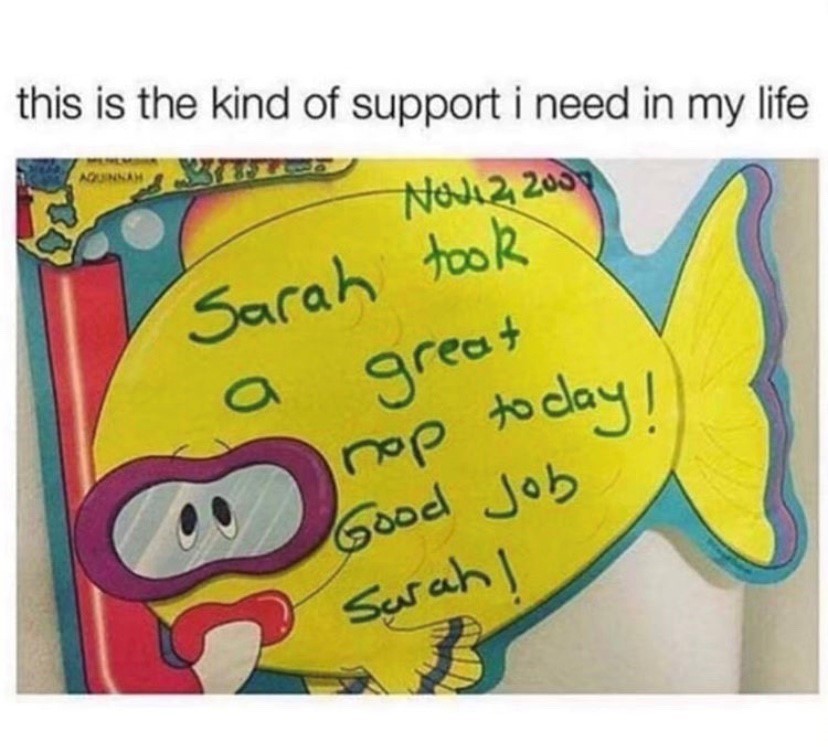 |
| 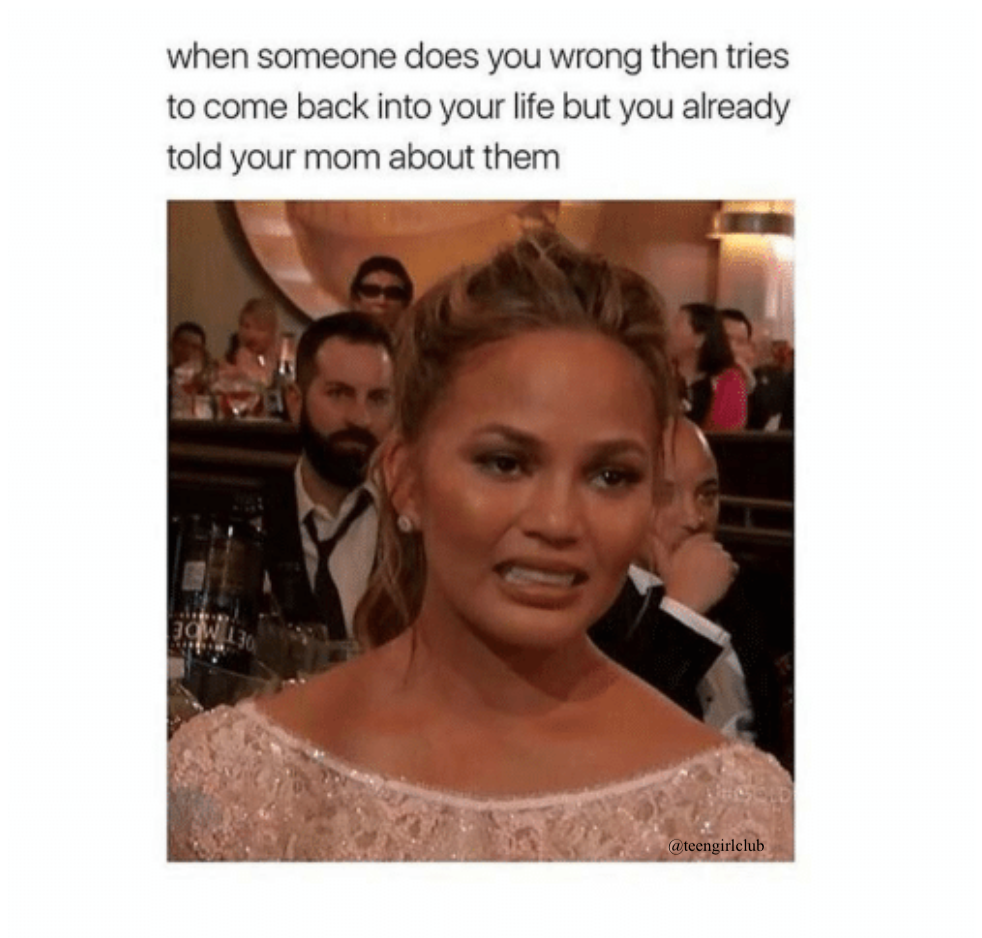 |
| 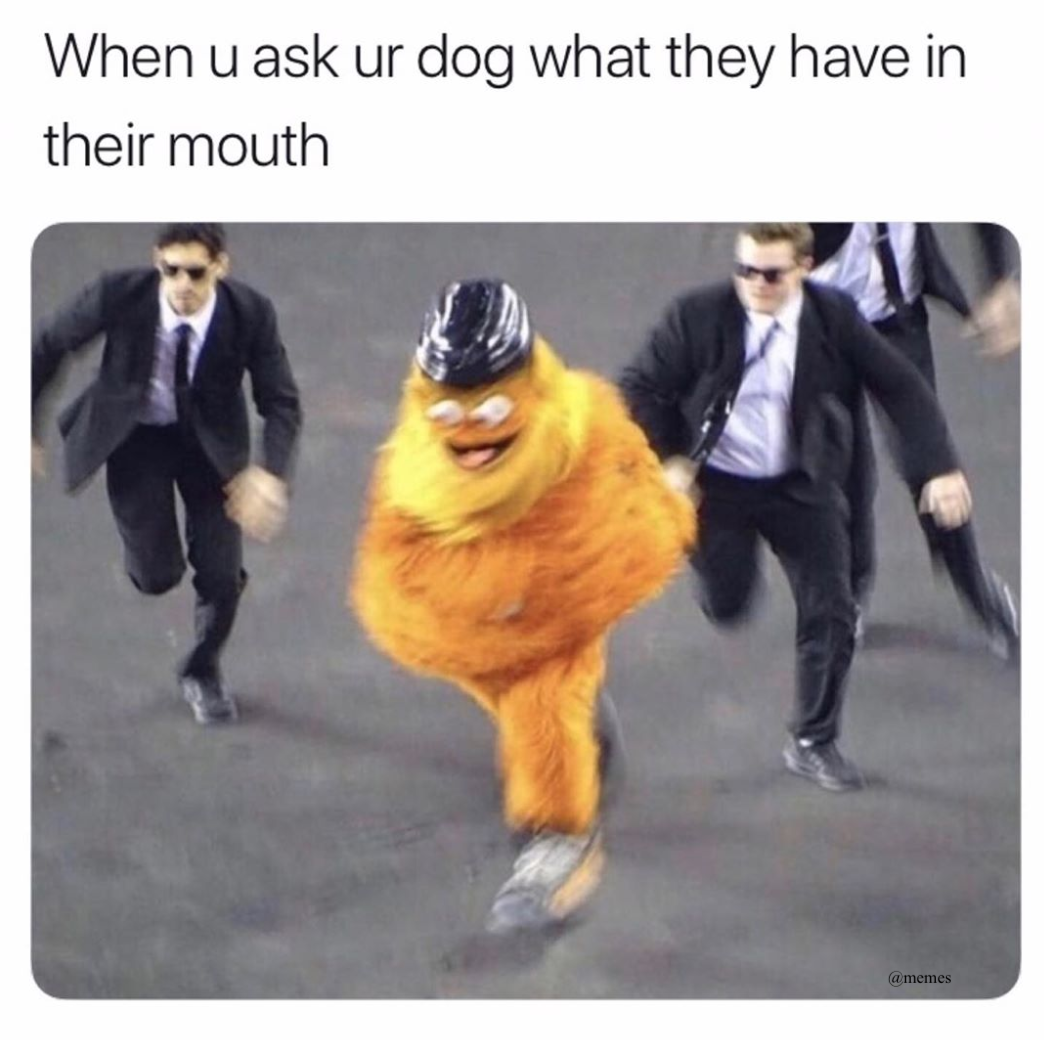 |
| 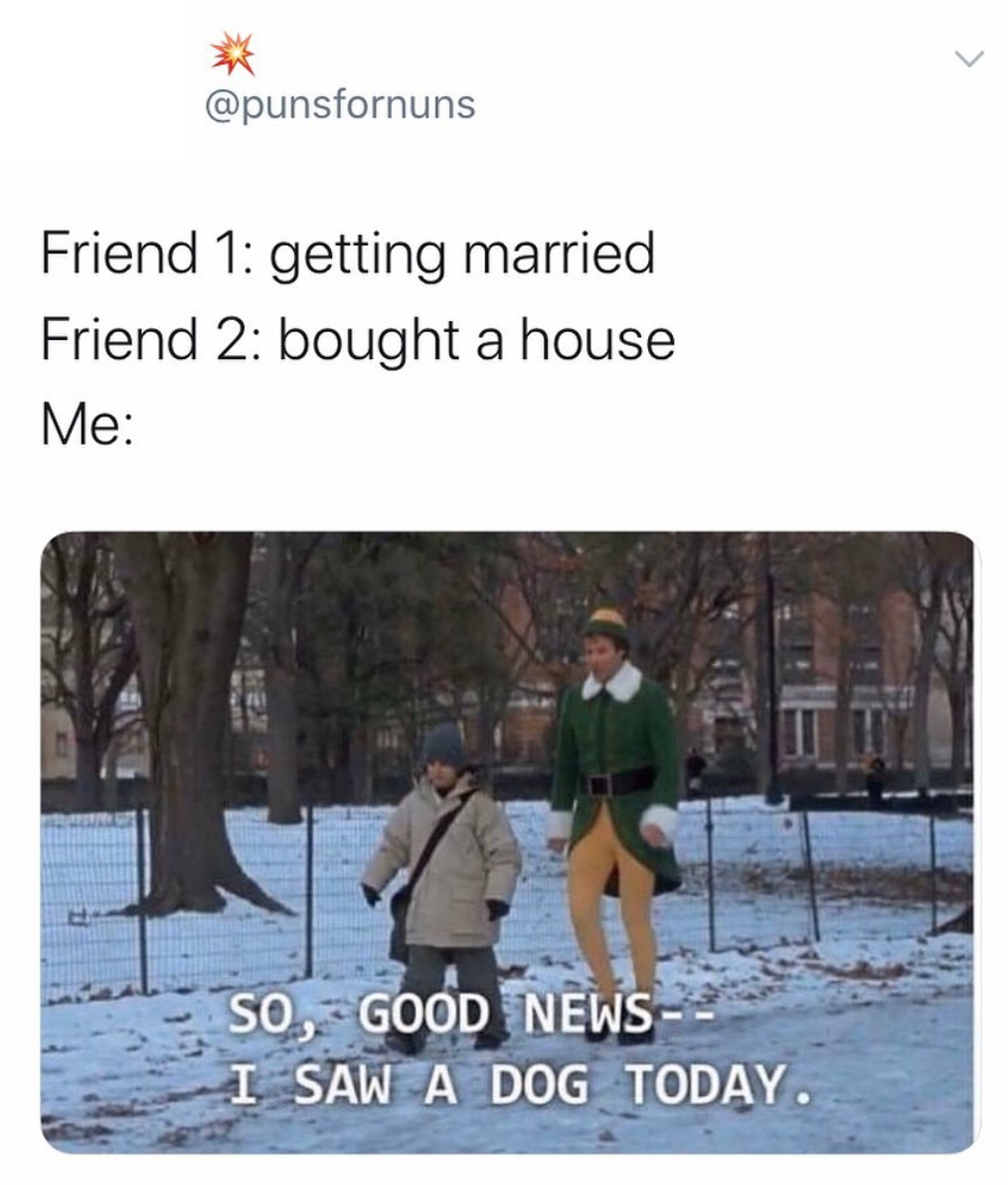 |
| 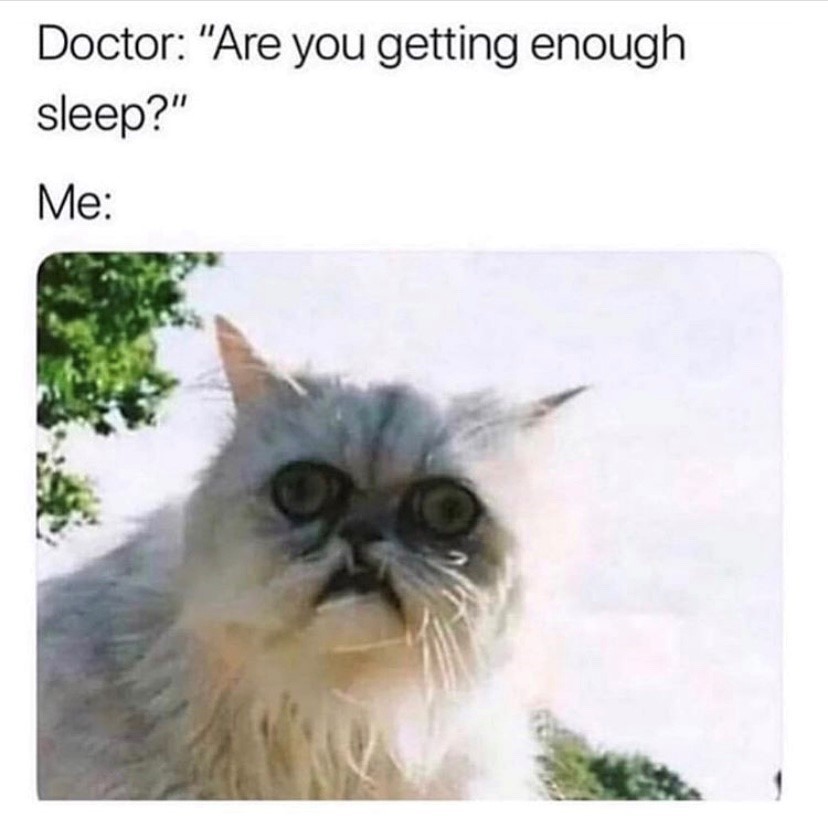 |
| 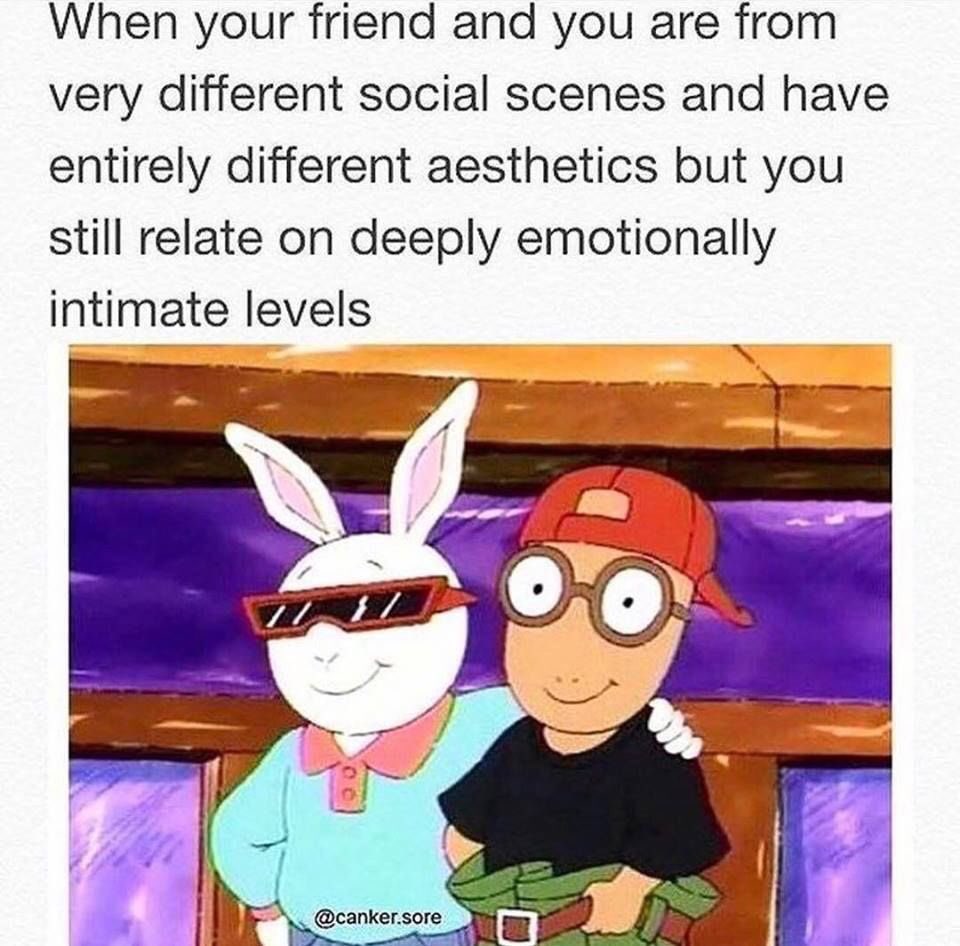 |
| 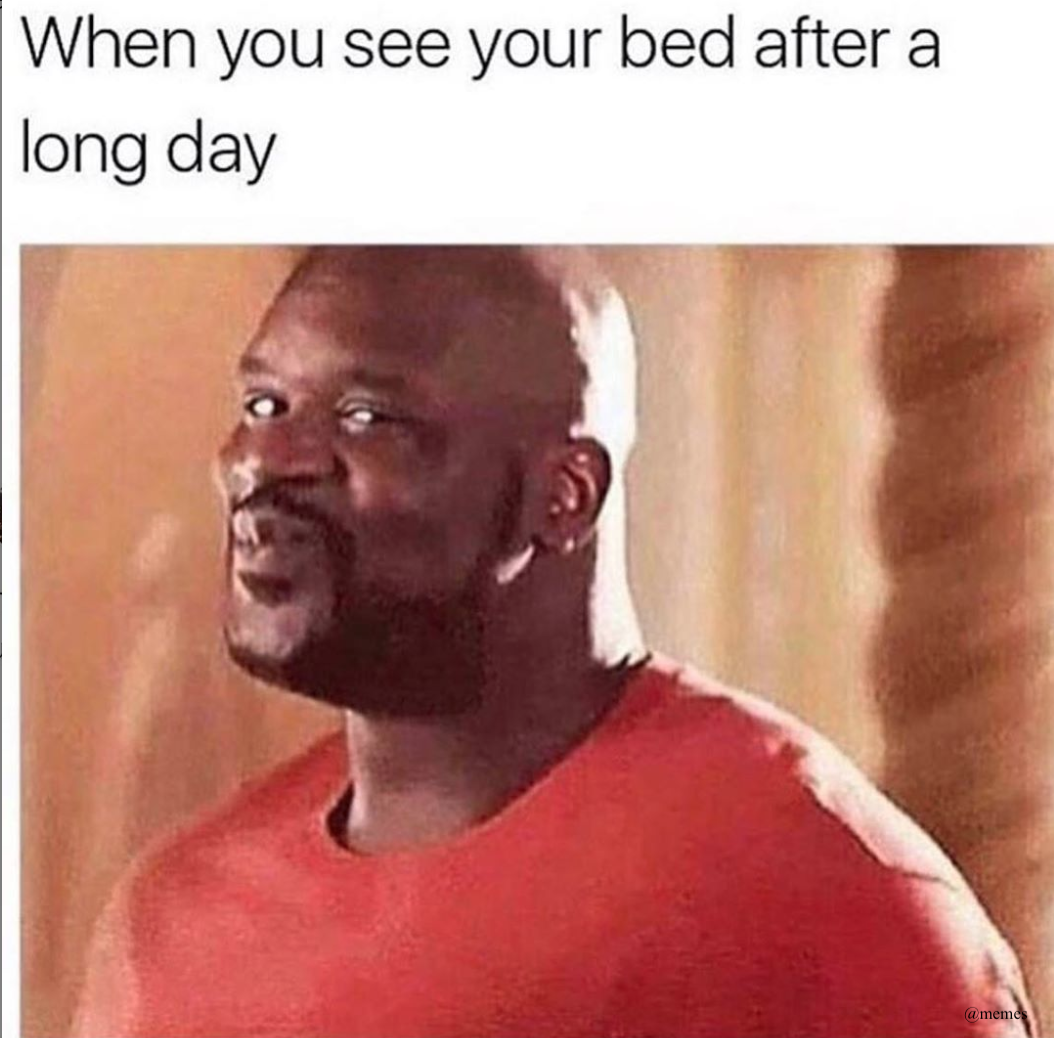 |
| 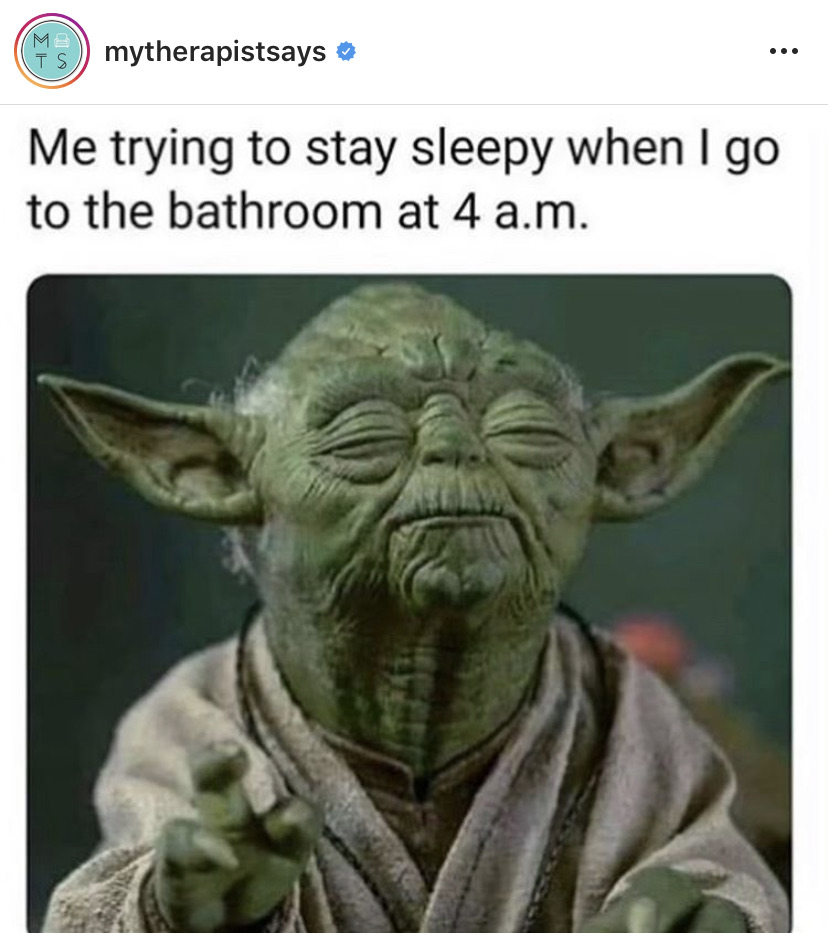 |
| 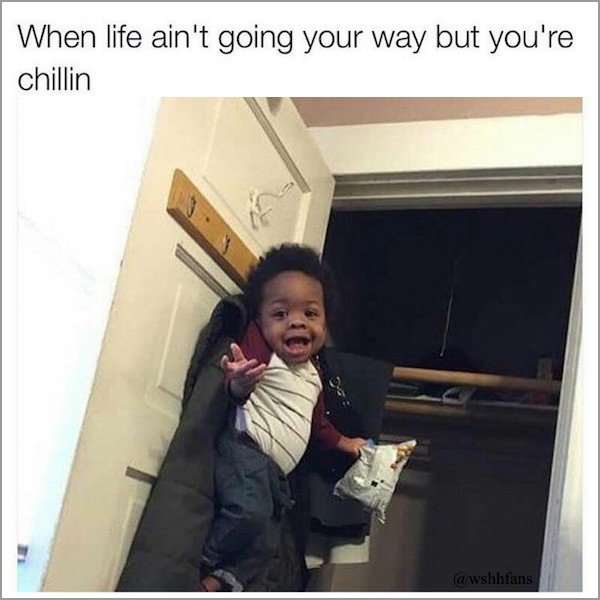 |
| 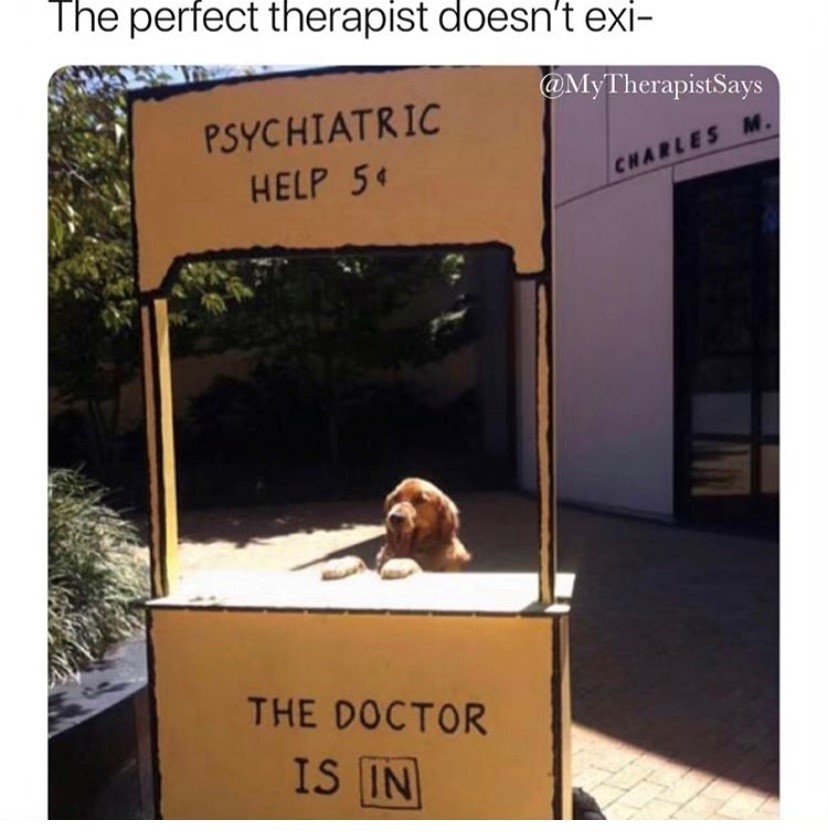 |
| 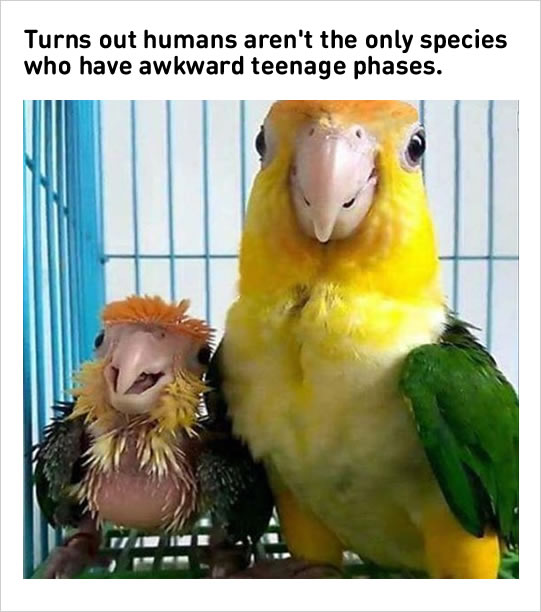 |
| 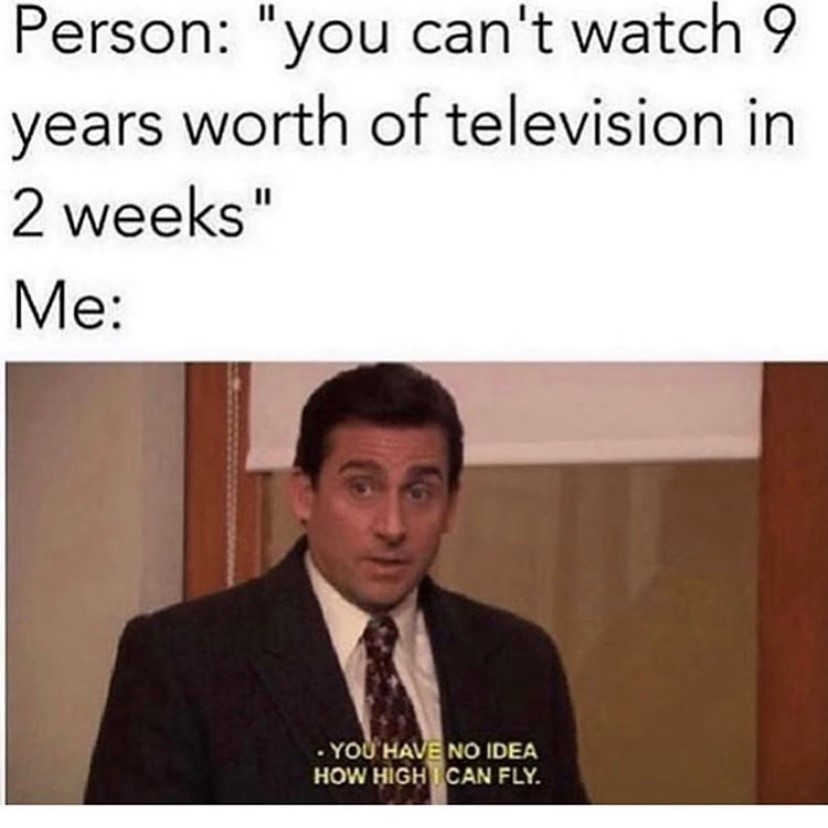 |
| 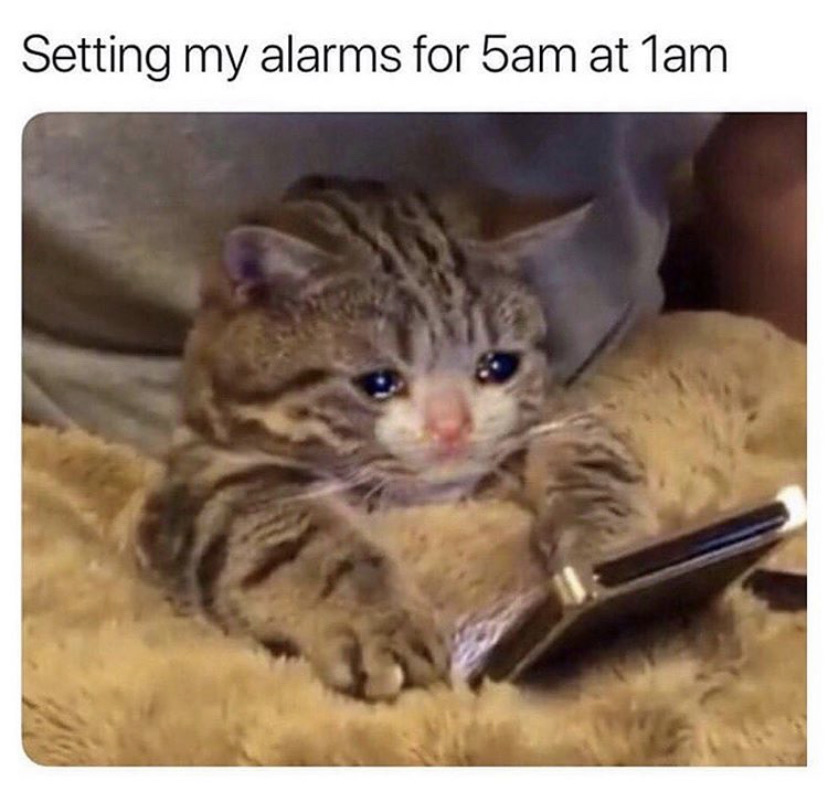 |
| 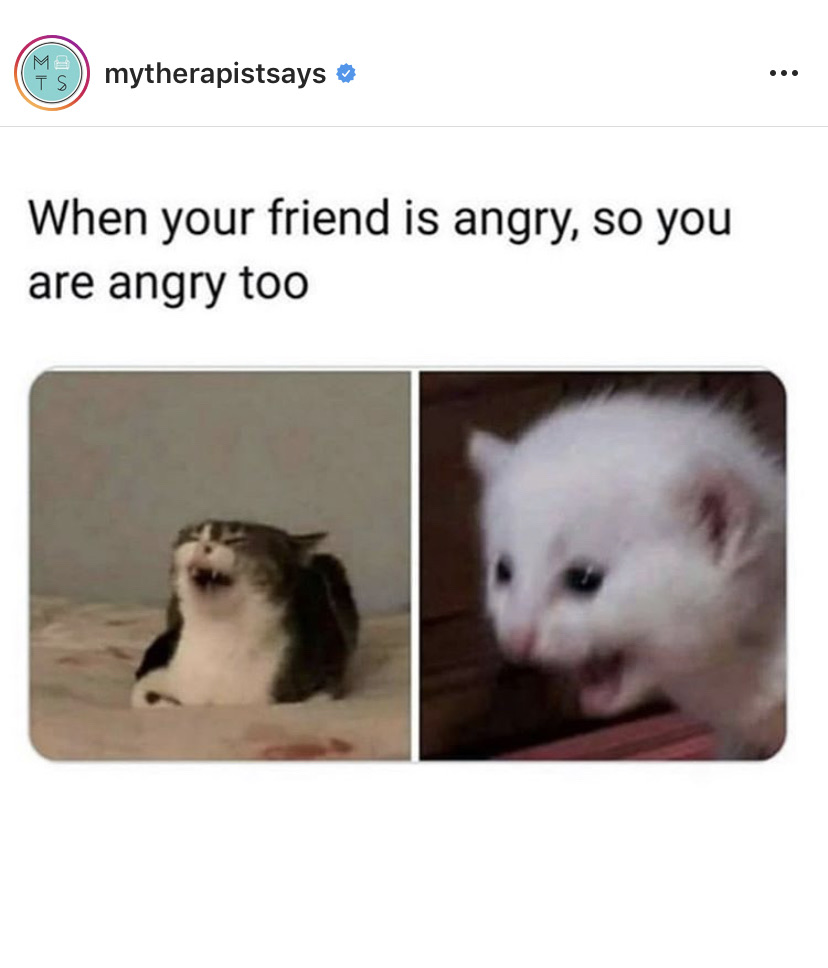 |
| 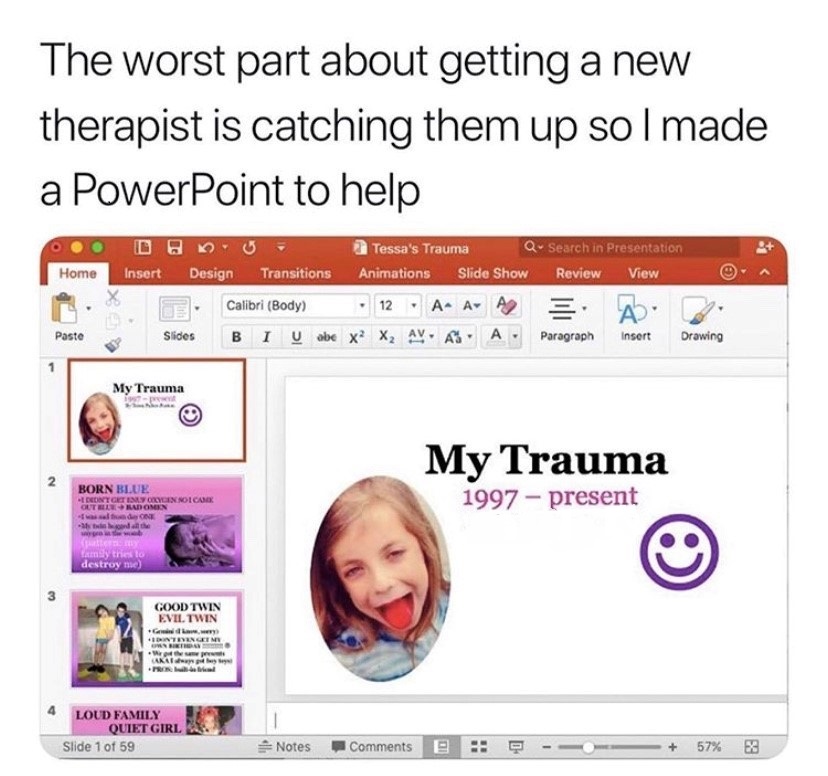 |
| 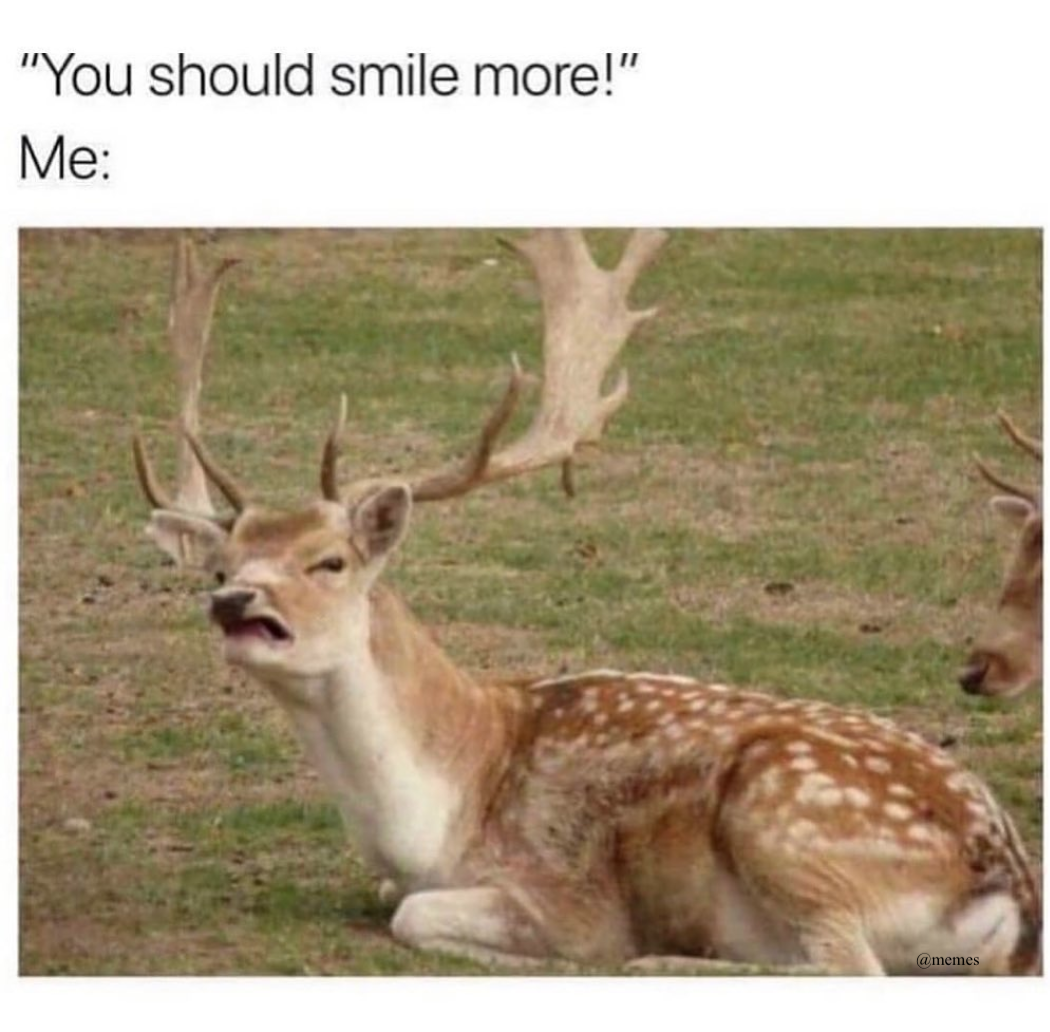 |
| 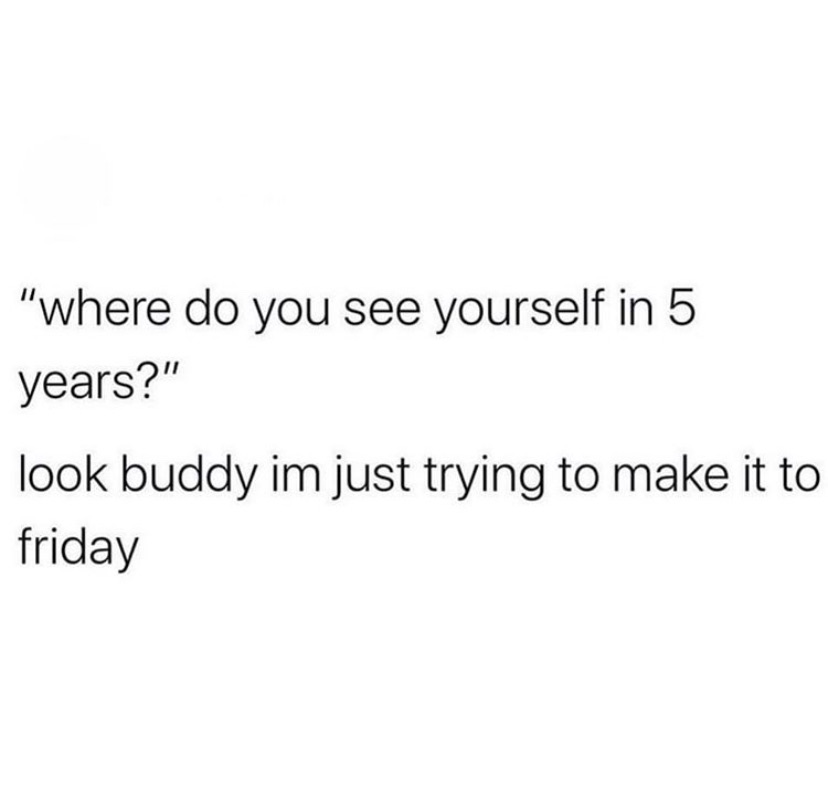 |
| 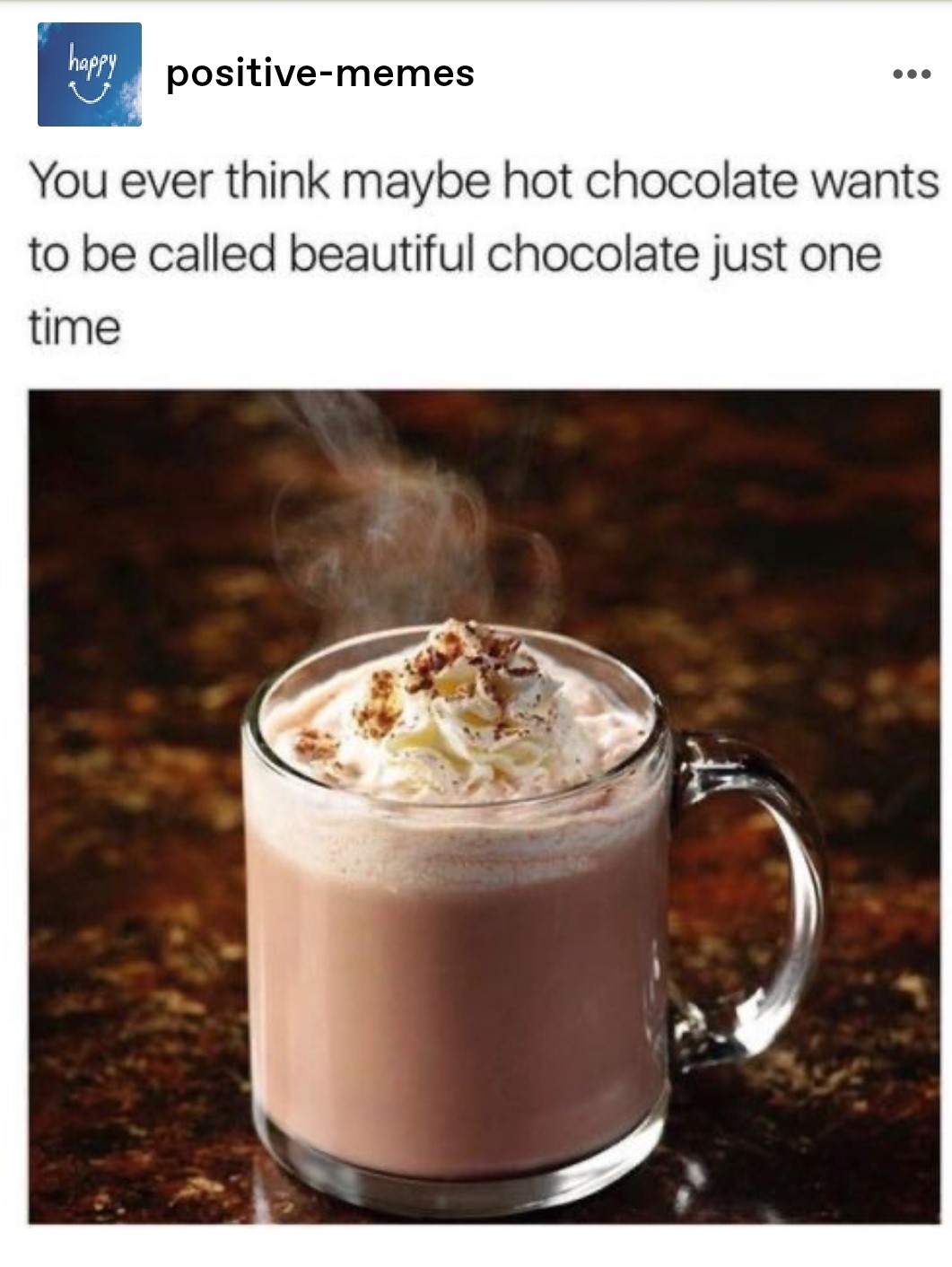 |
| 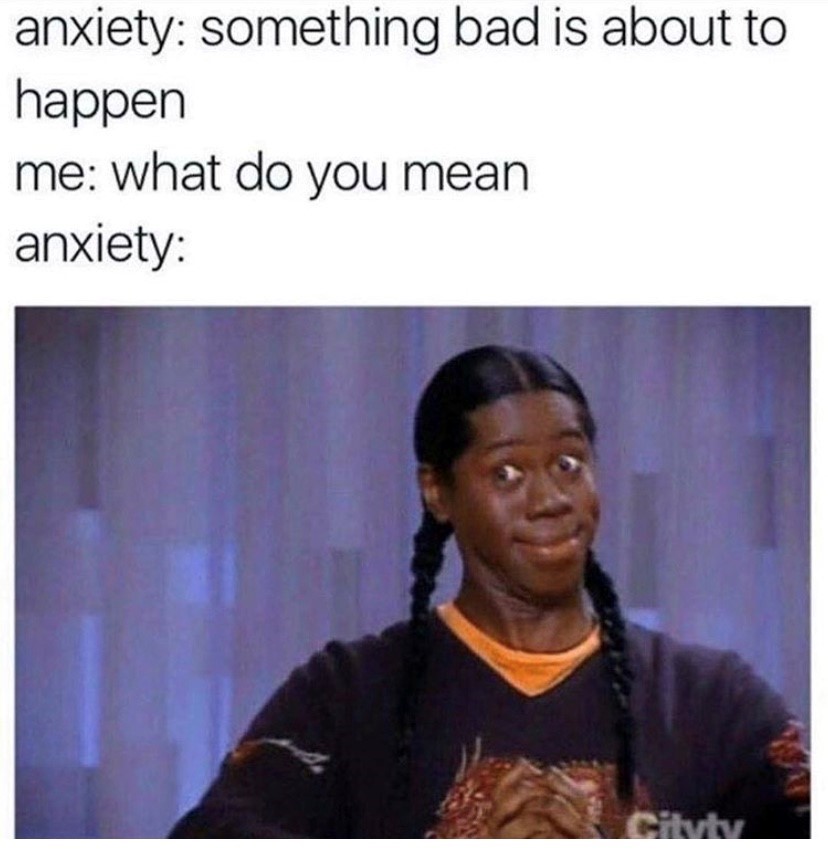 |
| 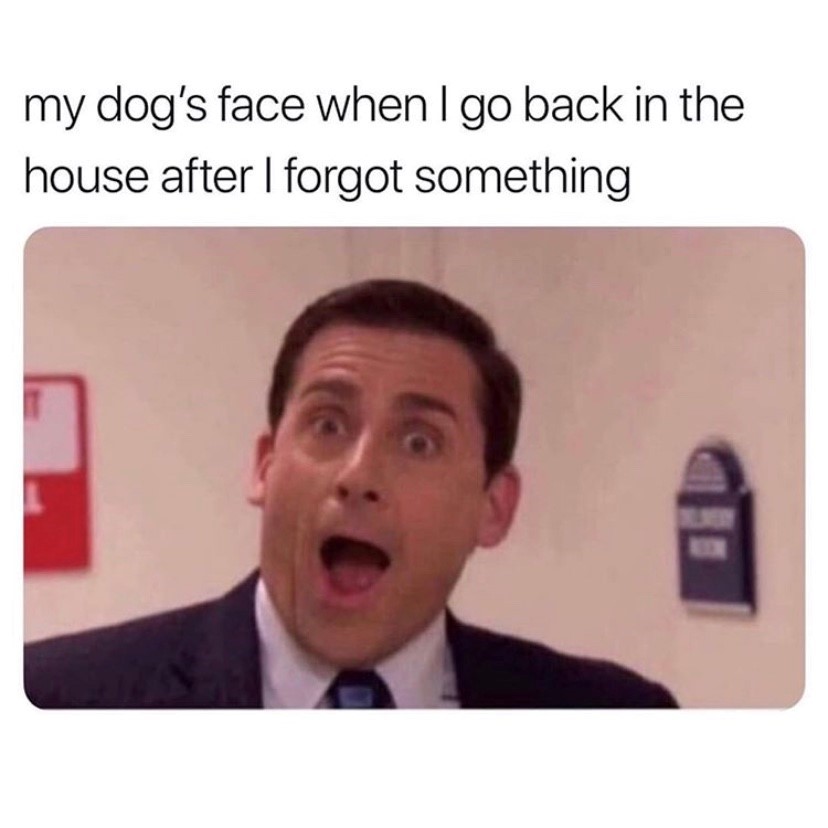 |
| 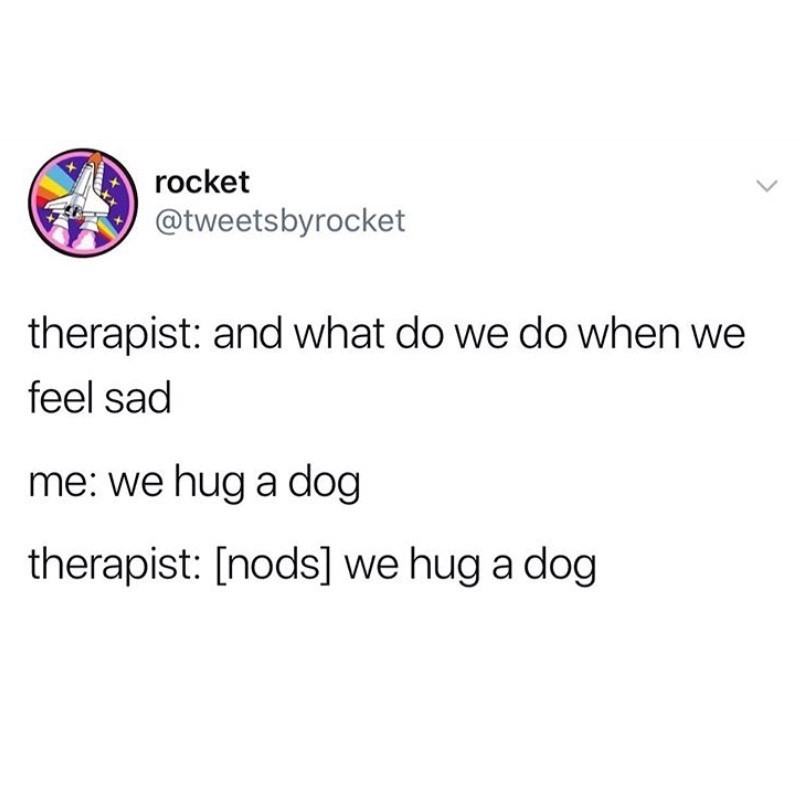 |
| 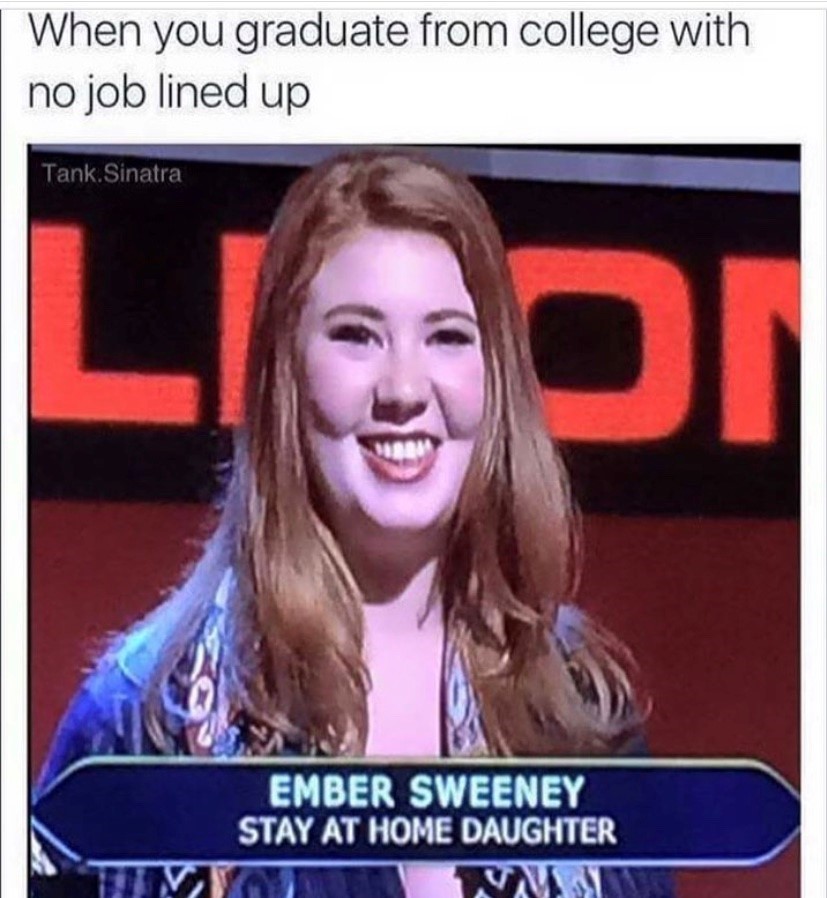 |
| 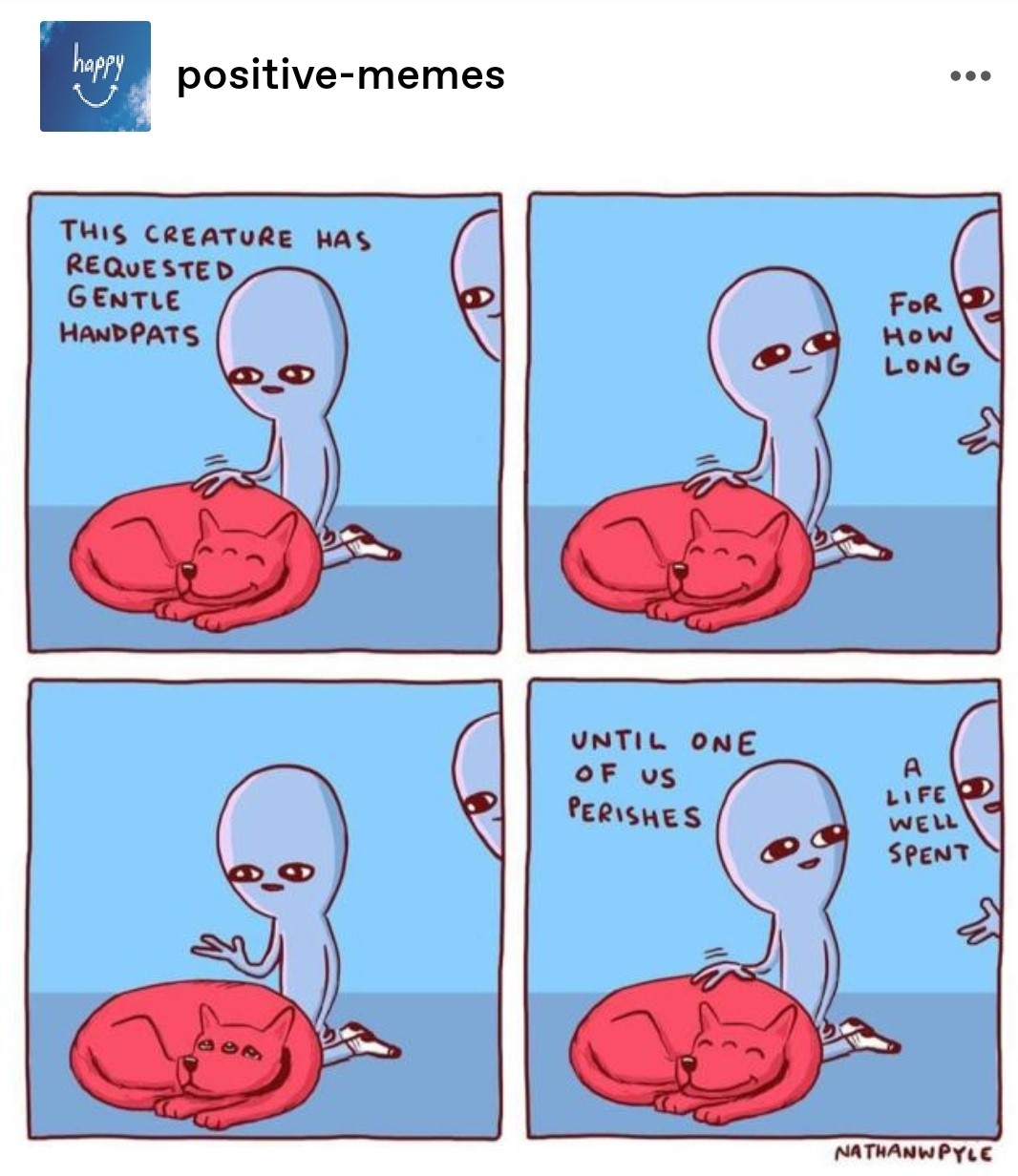 |
| 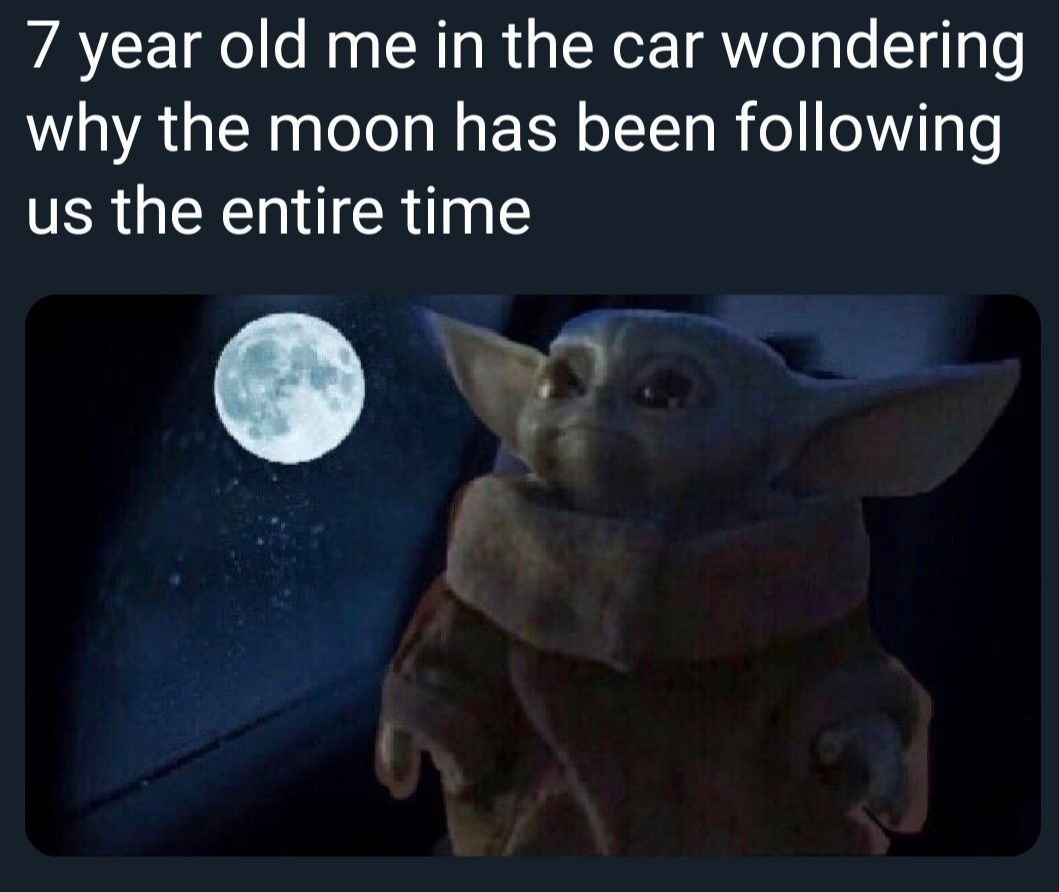 |
| 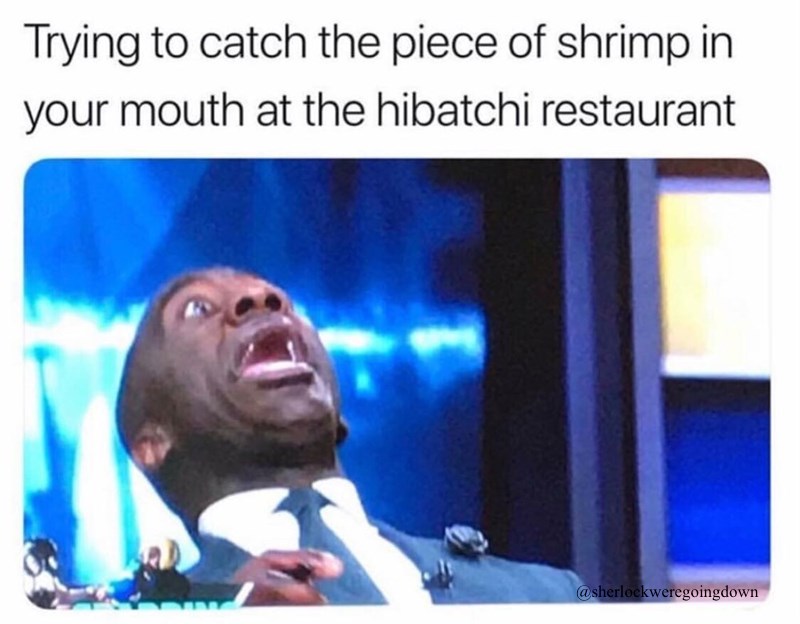 |
| 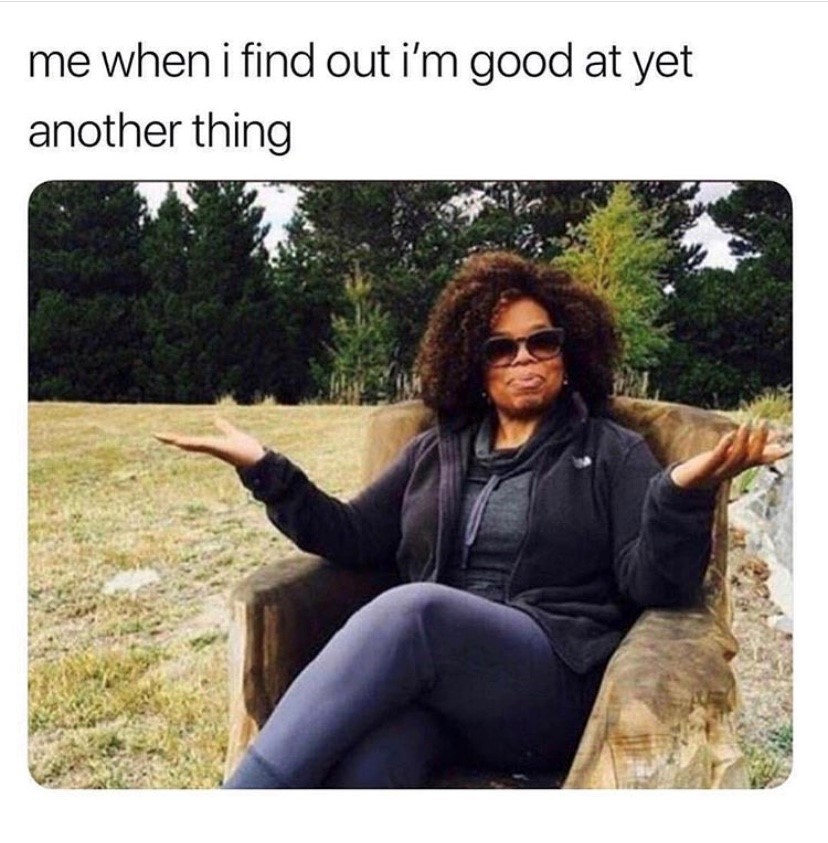 |
| 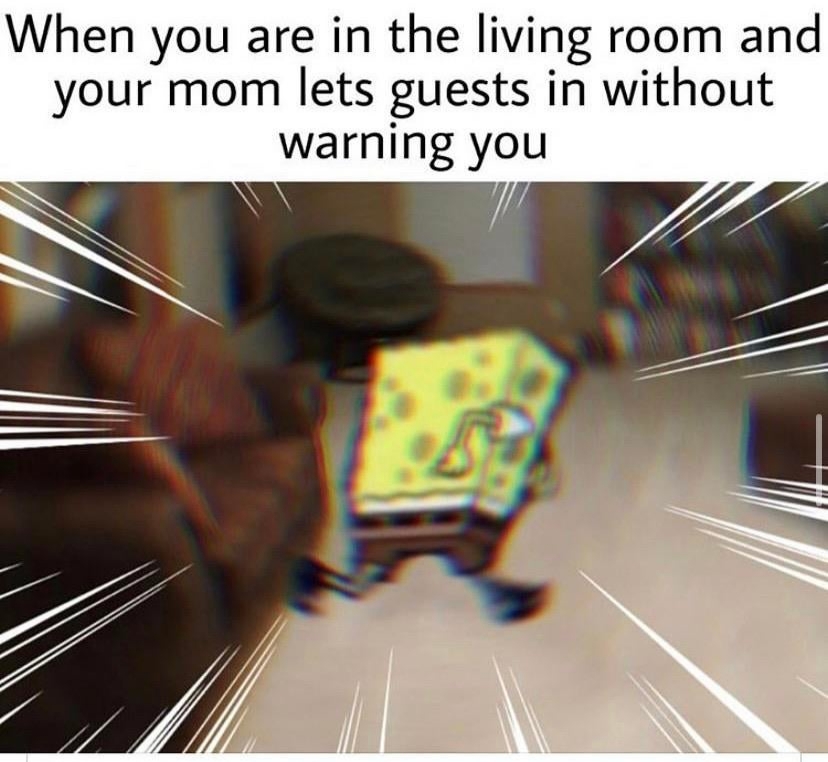 |

Supplement: Multimedia Appendix 2 [file resprot_v10i10e32789_app2.docx]

**Multimedia Appendix C: Altruistic Messages** *(21 total)*

| 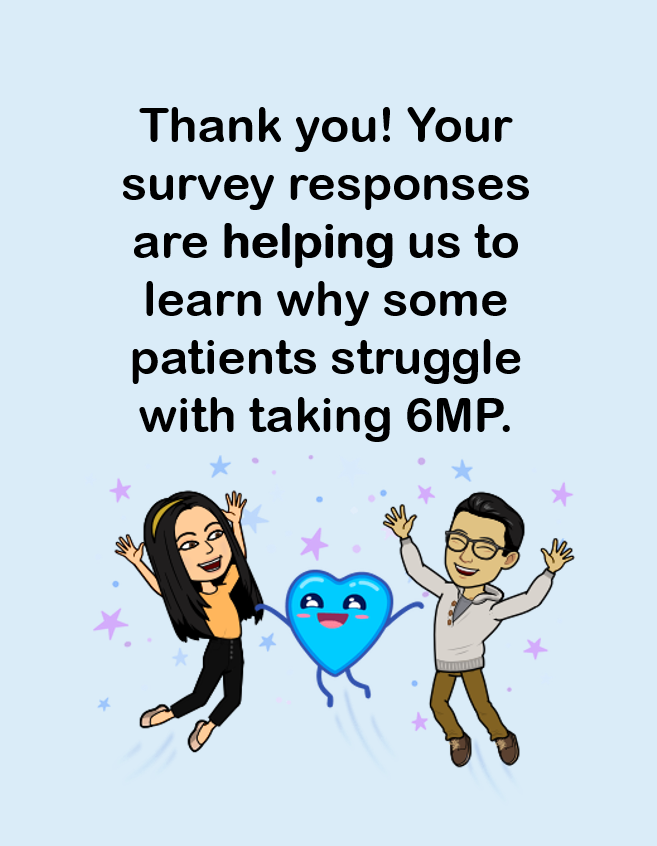 | 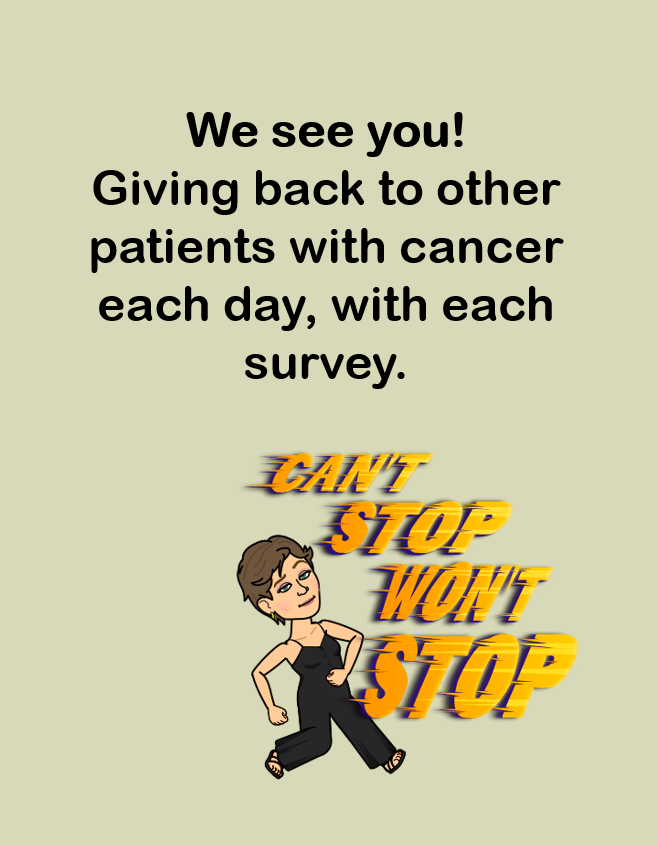 |
| --- | --- |
| 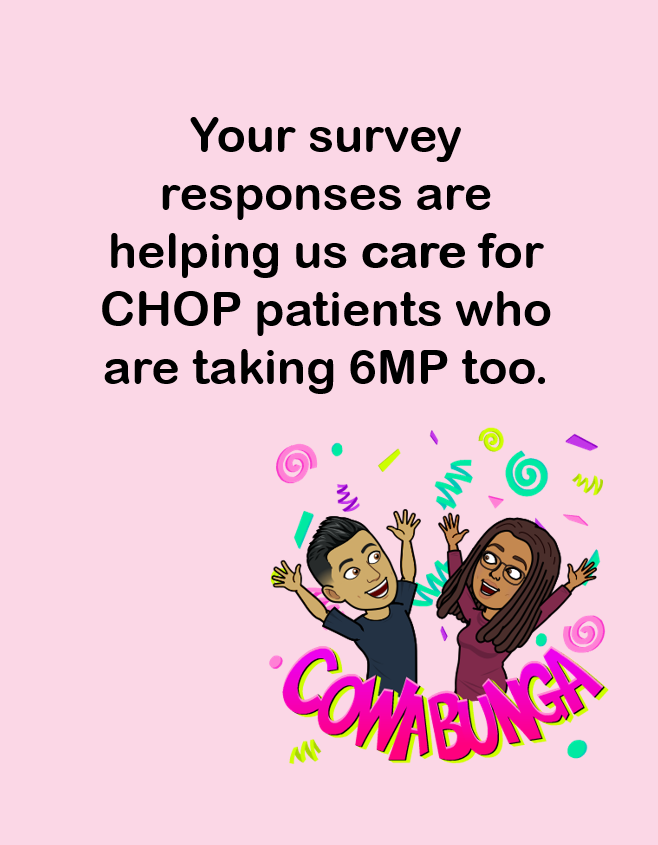 | 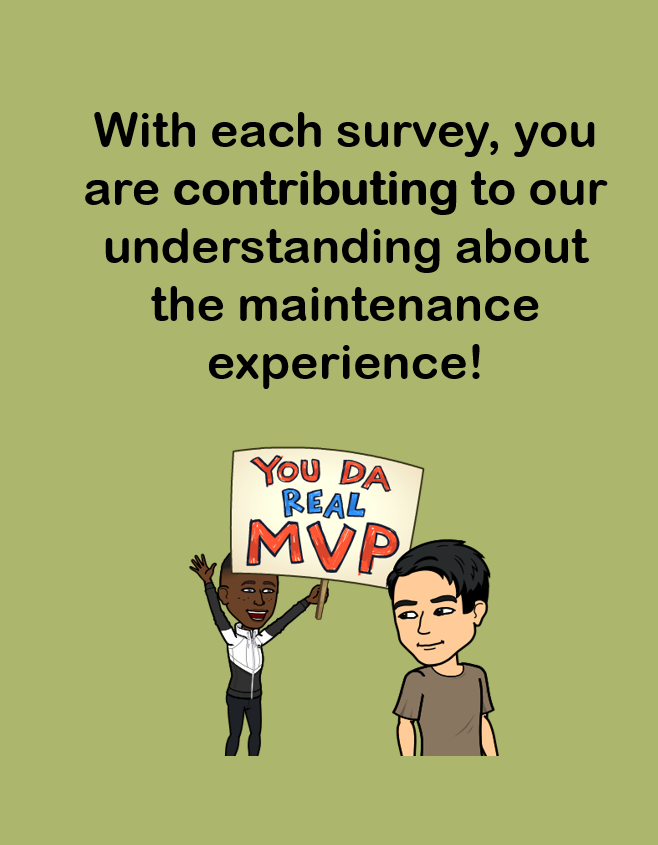 |
| 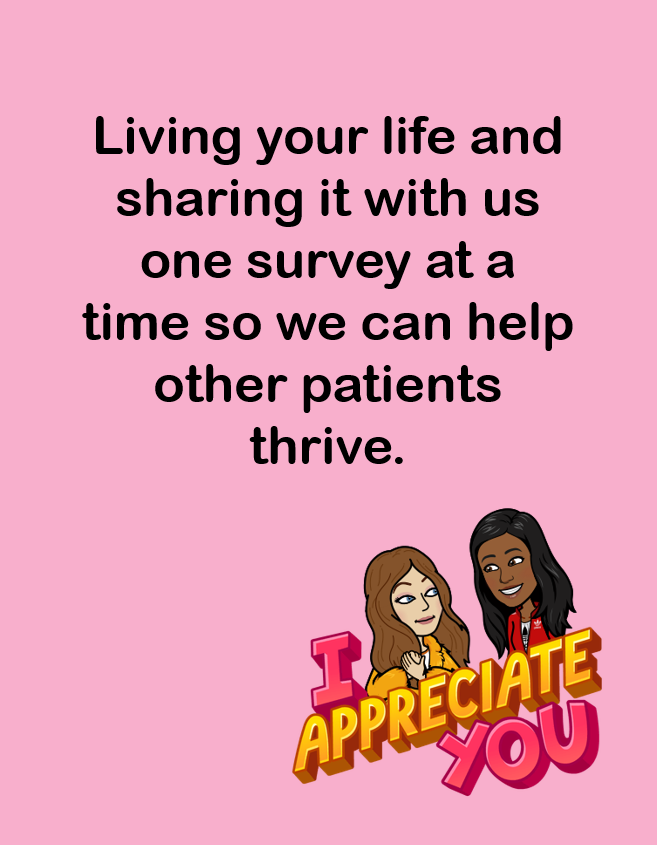 | 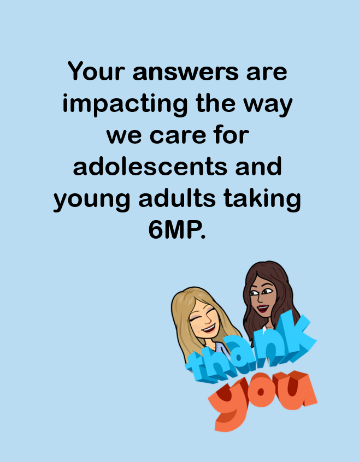 |
| 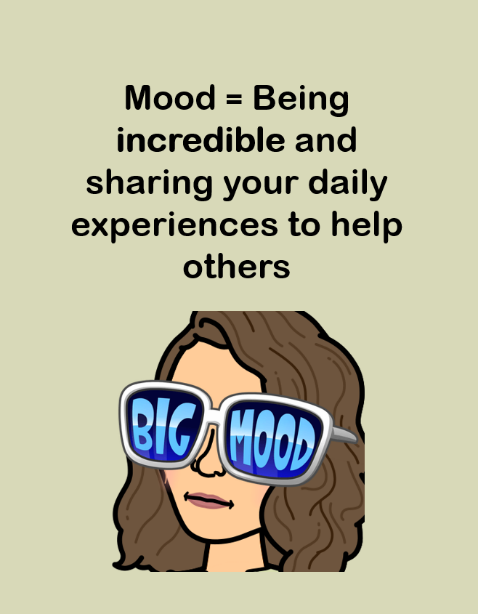 | 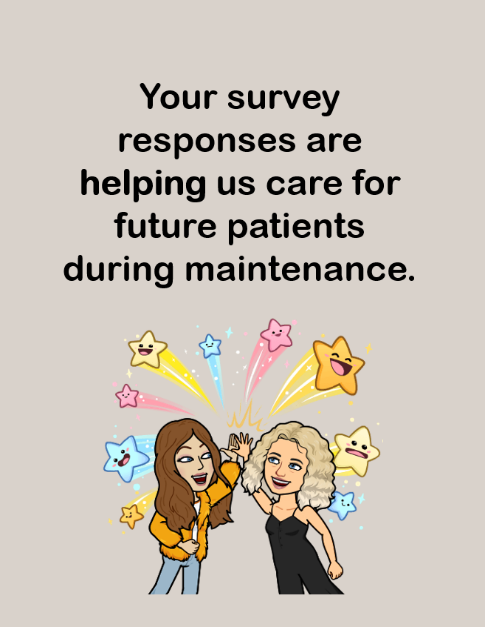 |
| 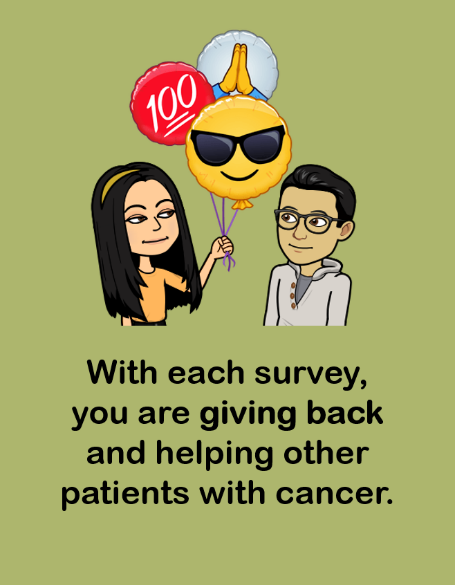 | 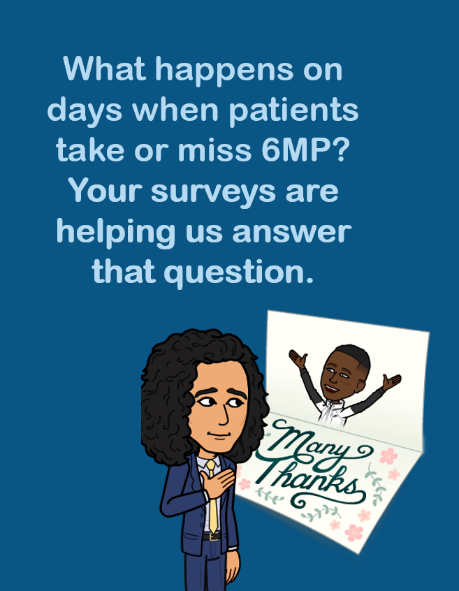 |
| 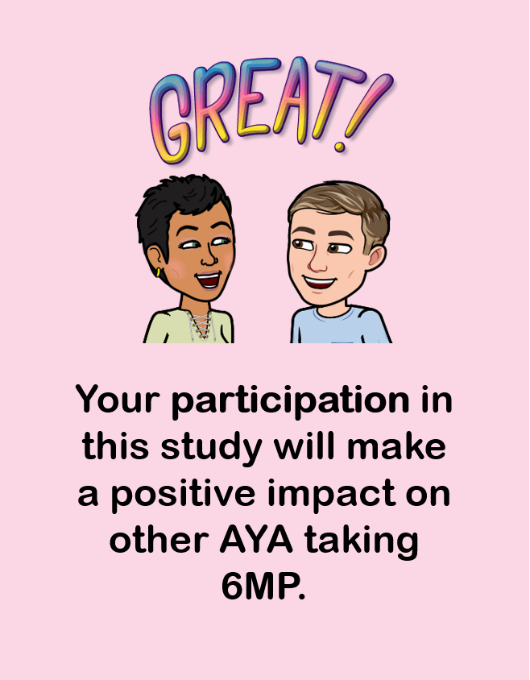 | 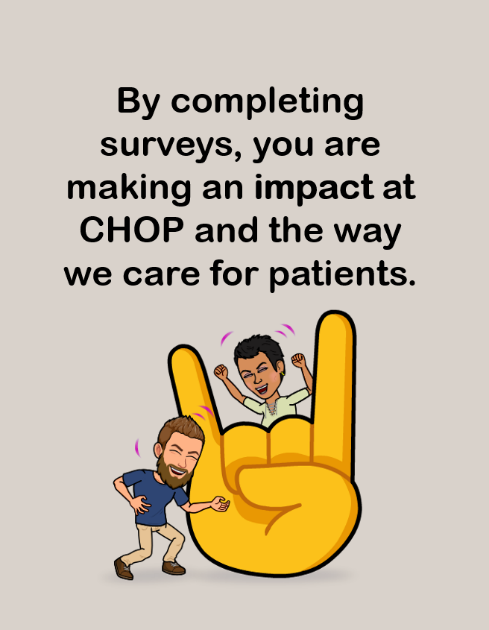 |
| 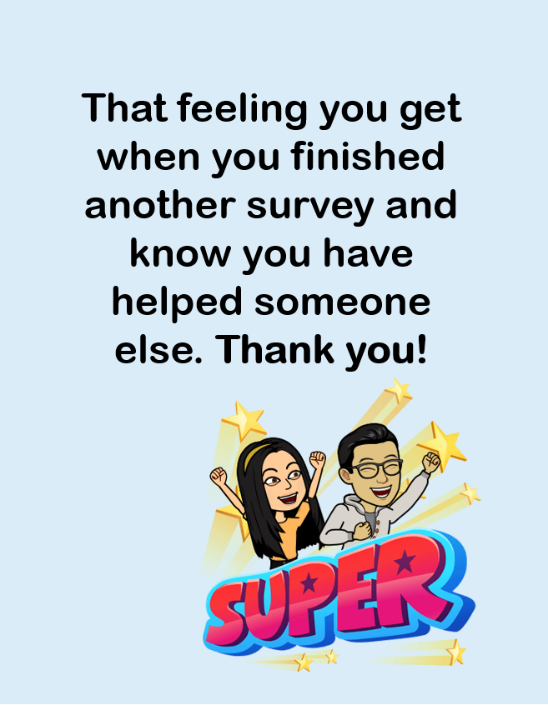 | 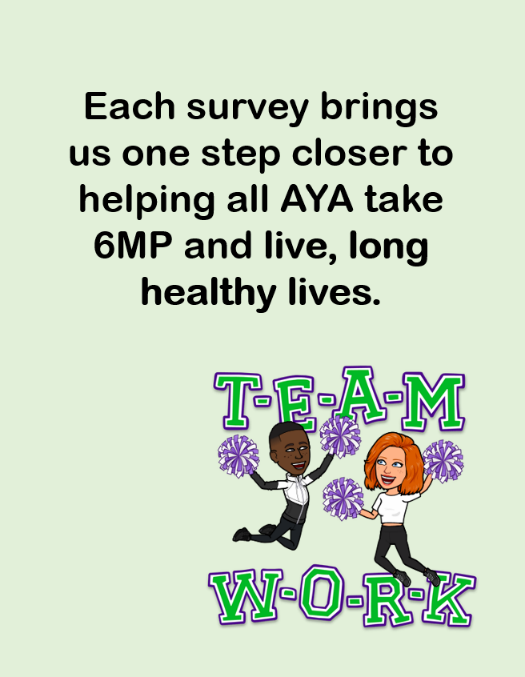 |
| 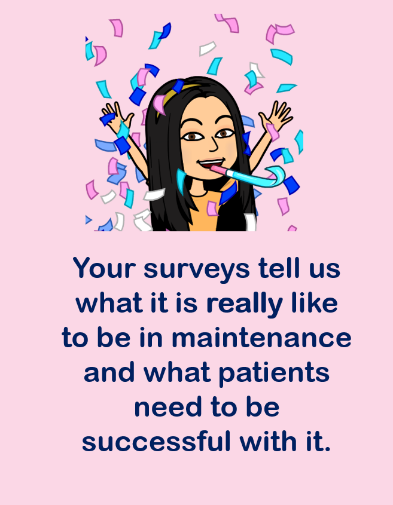 | 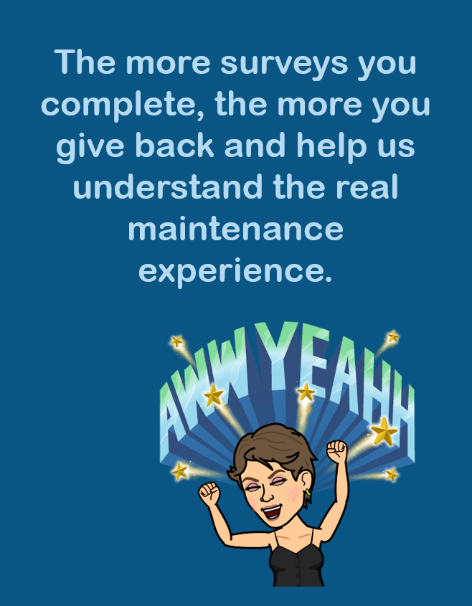 |
| 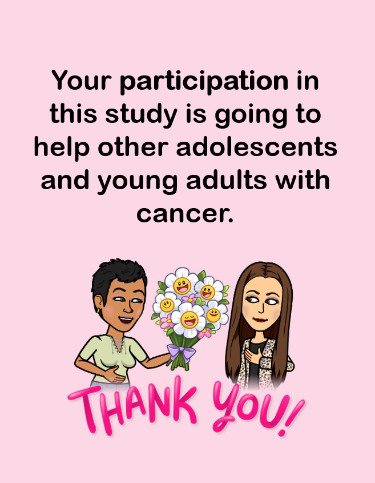 | 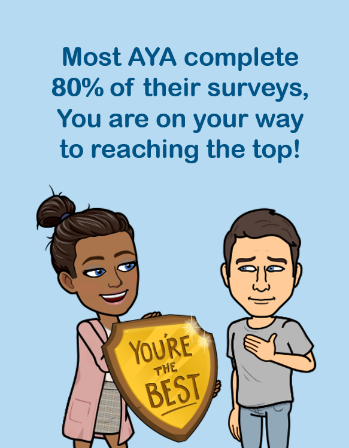 |
| 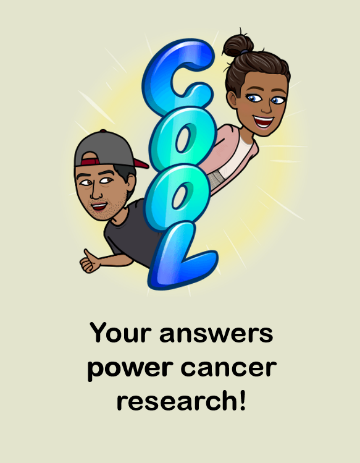 | 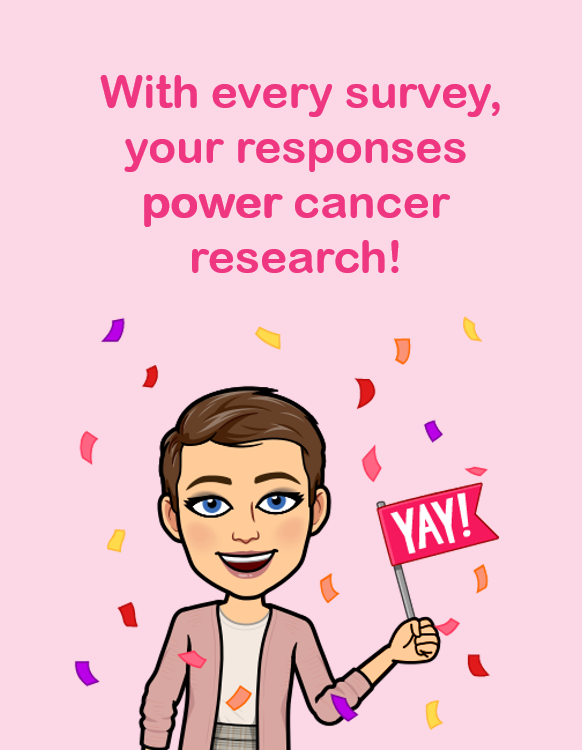 |
| 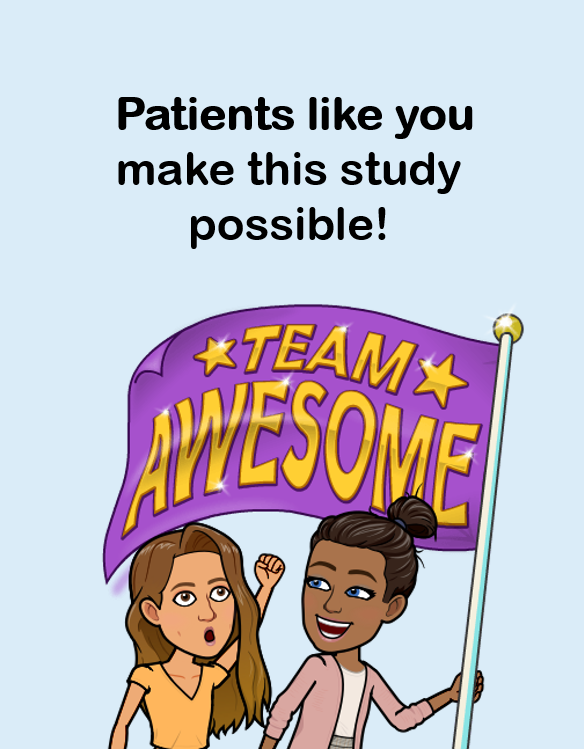 | |

Supplement: Multimedia Appendix 3 [file resprot_v10i10e32789_app3.docx]
