# Supplementary material for: Understanding Adolescent and Young Adult 6-Mercaptopurine Adherence and mHealth Engagement During Cancer Treatment: Protocol for Ecological Momentary Assessment
Source: JMIR Res Protoc. 2021 Oct 22;10(10):e32789. doi: 10.2196/32789 (PMC8571686; doi:10.2196/32789)
Supplement: Multimedia Appendix 4 [file resprot_v10i10e32789_app4.docx]

**Multimedia Appendix D: Animal Fun Facts** *(70 total)*

| **Animal** | **Fun Fact** |
| --- | --- |
| **Level 1: Aquarium** | |
| Clown Fish | Did you know the animators of "Finding Nemo" studied dogs’ facial expressions and eyes to animate the fishes’ expressions? |
| Butterfly Fish | Did you know there are 114 species of butterfly fish? |
| Gold Fish | Did you know gold fish can recognize faces? |
| Tiger Barb | Tiger barbs can swim fast and they like to spend their time chasing other tiger barbs. |
| Squid | Some species of squid can swim at speeds up to 25 miles per hour, as fast as some sharks. |
| Octopus | Did you know an octopus has 3 hearts and blue blood? |
| Angelfish | Did you know if an angelfish's partner dies, then it has no interest in finding another partner? |
| Crab | A crab's eyestalks can move in different directions which allow it to see all around? |
| Starfish | Starfish have 2 stomachs and can regenerate their arms. |
| Carp | Did you know carp fish can grow to be huge? The largest carp on record weighs 222lbs. |
| Electric Fish | Electric fish have large brain-to-body size ratios and can use their electric discharge to communicate social status. |
| Discus Fish | Discus fish are perhaps the most beautiful tropical fish and known as the King of the Aquarium. |
| Betta Fish | Most betta fish in aquariums are red and blue in color, but wild bettas are brown or green. |
| Sea Horse | Did you know the male sea horse bears the unborn young? This is rare in the animal kingdom. |
| **Level 2: Ocean** | |
| Blue Tang Fish | If you are a shark, never eat blue tang fish. Blue tang is Paracanthurus hepatus, a poisonous fish. |
| Jellyfish | Jellyfish have been on Earth for millions of years, even before dinosaurs! |
| Red Crab | A crab's shell protects the soft abdomen and hydrates the gills so that it can breathe well. |
| Green Crab | Did you know a crab sometimes wants another crab's shell, then a shell fight triggers? |
| Anchovy | Anchovies are found in temperate waters, and are rare in very cold or very warm seas. |
| Bubbles | Bubbles is in the fishtank gang. Like a playful puppy obsessed with a toy, he is enamored with bubbles. |
| Salmon | Did you know salmon can jump two yards into the air? |
| Deep Undersea Fish | Deepsea fish live below 1000 meter of the sea level where no sunlight can reach. They can generate light biologically. |
| Sword Fish | Sword fish can swim up to 60 miles per hour! |
| Shark | Most sharks species will drown if they stop moving. |
| Dolphin | Dolphins communicate with each other by clicking, whistling and other sounds. |
| Whale | Did you know that many whales are toothless? |
| Stingray | Ancient Greeks extracted venom from stingray spines and used it as an anesthetic. |
| **Level 3: Tundra** | |
| Arctic Hare | During winter, artic hare's white fur allows them to blend into the snow. In spring, their coat turns blue-gray to match the landscape. |
| Coyote | Dogs run with their tails up while coyotes run with their tails down. |
| Baby Penguin | When penguins begin to hatch, the chick will chip away from the shell until they can break out. |
| Brown Bear | Brown bears can be found in many habitats, from the fringes of deserts to high mountain forests and ice fields. |
| Blue Jay | Blue jays warn other birds of predators by imitating the sound of hawks! |
| Duck | The King Eider sea duck lives in the Arctic and dives deep down to the ocean floor to find food! |
| Big Penguin | The earliest known penguin fossil was found in 61.6 million-year old rock. Some fossil penguins were much larger than any penguin today, reaching 4.5 feet tall! |
| Grey Owl | Arctic owls can fly at speeds up to 50mph! |
| Rabbit | The Snowshoe Rabbit has giant hind feet (like skies) that prevent it from sinking into the snow! |
| Polar Bear | Polar bears have black skin and although their fur appears white, it is actually transparent. |
| Stalagmite | Stalagmites grow up from the floor, usually caused by water dripping from above that quickly freezes. |
| Grey Husky | Living in the Arctic Circle, the Arctic wolf spends five out of twelve months in total darkness. |
| Mountain Goat | Both male and female adult mountain goats have long hair under their throat forming a "beard." |
| Yeti | In 2019, Indian mountaineers uncovered a large sets of footprints in the northeastern Himalaya snow which led them to believe the prints belonged to Yeti. |
| Dark Grey Wolf | A wolf’s howl is the loudest when in harmony with others. Yet, a single wolf's howl can be heard up to 10km. |
| Snow Globe | The first known record a snow globe went on display at the Paris Universal Expo in 1878. |
| Snowman | The record for world's largest snowman stands at a whopping 122 ft tall! |
| Pink Earless Seals | When they are on the land, they live in huge colonies with over one thousand other seals! |
| Reindeer | Male reindeer lose their antlers in November, but females keep theirs much longer. |
| Snow Bunting (Small Bird) | Snow buntings have been called "snowflakes" because flocks look like snowflakes as they swirl through the air and then settle on winter fields. |
| Snowy Owl | The Arctic summer forces snowy owls to hunt by daylight. Unlike most owls that are nocturnal, snowy owls are diurnal. |
| **Level 4: Rainforest** | |
| Black Panther | The black panther is often called 'the ghost of the forest'. Its dark coat helps it hide and easily stalk prey at night. |
| Brown Spider Monkey | A brown spider monkey's tail has a patch of skin at the end which allows them to grip branches. |
| Toucan | Toucans aren't very good at flying. They travel through the trees by hopping! |
| Lizard | The Anolis lizard, with brilliant blue skin, is so elusive and rare that scientists can't even estimate its population. |
| Gorilla | Gorillas are very intelligent. They can use simple tools and learn sign language. |
| Macaw | Macaws usually live for around 60 years! |
| Black Spider Monkey | When different troops of spider monkeys meet, they hug each other to express greetings and avoid confrontation and potential aggression. |
| Butterfly | The blue morpho is among the largest butterflies in the world, with wings spanning from five to eight inches |
| Cougar | Cougars can purr and growl, but they can't roar! |
| Owl | Owls can rotate their necks 270 degrees. |
| Red Howler Monkey | Red howler monkeys are the loudest monkeys in the world! Their sound can carry for 20 miles! |
| Orchid | According to the fossil evidences, orchids have existed on the planet around 100 million years. |
| Jaguar | Jaguars once roamed all the way to the US-Mexico border. Today, almost all jaguars are found in the Amazon river basin. |
| Sparrow | The song of Pectoral Sparrow is high and thin. It has been described as "insect-like .. an extremely high, thin, buzzy chit-tic-tzzzzzz, tzzzzzz, tzzzzz" |
| Snake | Snakes don’t have eyelids, so are unable to close their eyes. They can’t blink and they sleep with their eyes open. |
| Venus Flytrap | It takes roughly 10 days for a venus flytrap to completely digest an insect. |
| Ostrich | Ostriches formerly occupied Africa south of the rainforest belt. Today common ostriches prefer open land and are native to the savannas. |
| Squirrel | Squirrels have four front teeth which never stop growing throughout their lives. |
| Vulture | The king vulture is one of the larger species of vulture (up to 32 inches long). All of the other species vultures are quick to move aside for the "king." |
| Flock (Bird) | Some species of birds take their first migration on their own, never before seeing where they are supposed to go, they just know! |
