# Supplementary material for: Understanding Adolescent and Young Adult 6-Mercaptopurine Adherence and mHealth Engagement During Cancer Treatment: Protocol for Ecological Momentary Assessment
Source: JMIR Res Protoc. 2021 Oct 22;10(10):e32789. doi: 10.2196/32789 (PMC8571686; doi:10.2196/32789)
Supplement: Multimedia Appendix 5 [file resprot_v10i10e32789_app5.pdf]

SUMMARY STATEMENT

(Privileged Communication)

PROGRAM CONTACT:
SERGEY RADAEV
240-276-6466
sradaev@mail.nih.gov

Release Date: 04/03/2019
Revised Date:

Application Number: 1 K08 CA241335-01

Principal Investigator

PSIHOGIOS, ALEXANDRA

Applicant Organization: CHILDREN'S HOSP OF PHILADELPHIA

Review Group: NCI-J
Subcommittee J - Career Development

Meeting Date: 02/28/2019
Council: MAY 2019
Requested Start: 07/01/2019
RFA/PA: PA18-372
PCC: AHTR
Dual IC(s): HD, MH

Project Title: Using Real Time Mobile Health Approaches to Understand and Promote Oral Chemotherapy Adherence in Adolescents and Young Adults with Leukemia
SRG Action: Impact Score:28
Next Steps: Visit https://grants.nih.gov/grants/next_steps.htm
Human Subjects: 30-Human subjects involved - Certified, no SRG concerns
Animal Subjects: 10-No live vertebrate animals involved for competing appl.
Gender: 1A-Both genders, scientifically acceptable
Minority: 1A-Minorities and non-minorities, scientifically acceptable
Age: 1A-Both Children and Adults, scientifically acceptable

Project Year	Direct Costs Requested	Estimated Total Cost
1	128,125	138,375
2	124,512	134,473
3	126,964	137,121
4	135,228	146,046
5	133,395	144,067
TOTAL	648,224	700,082

1K08CA241335-01 Psihogios, Alexandra

RESUME AND SUMMARY OF DISCUSSION: In this NCI Mentored Clinical Scientist Research Career Development Award (K08) application, the Principal Investigator (PI), Dr. Psihogios proposes to use real time mobile health methodologies to examine and intervene on the time-varying contextual factors that influence daily oral chemotherapy adherence in adolescents and young adults (AYA) with acute lymphoblastic leukemia (ALL) . Dr. Psihogios is a mentor supported stellar candidate with excellent prior training in clinical pediatric psychology and a strong commitment to cancer control particularly in the field of adherence intervention. The candidate has strong record of peer-reviewed publications including publications in cancer in AYA. Dr. Psihogios has been awarded with several prestigious academic awards including American Cancer Society (ACS) postdoctoral fellowship and Mattie Miracle Cancer Foundation award. The candidate is supported by strong reference letters which speak highly of her motivation and commitment as well as potential for becoming an independent investigator. A career development plan (CDP) excellently integrated with research and mentoring plan is presented. The CDP with long term goal to become an independent clinician-scientist aiming to improve cancer treatment outcomes in AYA by targeting nonadherence is strongly supported by mentors. The candidate identified her knowledge gaps in areas of ecological momentary assessment and multi-level statistics; behavior change intervention development incorporating mHealth; clinical trials; and intervention dissemination and implementation and accordingly formulated appropriate training modules consisting of mentorship, didactic coursework and seminars/workshops. Research plan has strong scientific and clinical premise to meet the needs of AYA cancer patients and to enhance medication adherence outcomes. Research plan is overall well designed with theoretically grounded and innovative intervention. The candidate gave thoughtful consideration of potential pitfalls and alternative strategies while crafting the research plan. Research plan has strong potential to serve as a vehicle for research independence. An outstanding and committed mentoring and advisory team with complementary expertise in adaptive interventions, AYA psycho-oncology, development of mobile messages, ALL, statistics and ecological momentary assessment and adherence will mentor the candidate. A very clearly articulated mentor’s statement describing the quality and extent of the mentors’ proposed role in providing guidance and advice to the candidate; the areas that the candidate needed improvements; and plans for monitoring and evaluating the candidate’s progress toward independence is presented. Proposed research and career development of the candidate will take place in an outstanding research and training environment of the Children's Hospital of Philadelphia and a strong institutional commitment not contingent on this award is provided. Few weaknesses that were discussed are inadequate formal coursework in areas of cancer and health disparities; advanced survey, study design and statistical methods; ambitious nature of the research plan; and inadequate discussion of data collection, transfer , management and safety considerations in the application of ecological momentary assessment (EMA) in the proposed research. Strengths of the application outweigh weaknesses and this application is expected to deliver an overall high impact on career development of the candidate on the trajectory of an independent clinician scientist that improves health outcomes in AYA with cancer by targeting nonadherence.

DESCRIPTION (provided by applicant): Stagnant survival and relapse rates for adolescents and young adults (AYA) with cancer are partially attributed to a modifiable health behavior—suboptimal cancer treatment adherence. Yet, effective interventions are lacking. Aligned with priorities of the NCI and other federal agencies, the long-term objective of this K08 application is to help resolve disparities in cancer treatment outcomes in AYA by targeting nonadherence to life-saving cancer treatments. The goal of this proposal is to employ novel real time mobile health methodologies to examine and intervene on the time-varying, contextual factors that influence AYA adherence to an oral chemotherapy called 6-mercaptopurine, which must be taken daily in the maintenance phase to prevent relapse. In Aim 1, the PI and her mentorship team will determine the temporal associations between time-varying contextual factors (such as fatigue, motivation) and daily 6-mercaptopurine adherence. This Aim will utilize a 6-month intensive longitudinal design that employs bursts of mobile-based

ecological momentary assessment with AYA patients and their caregivers (n=30 pairs). In Aim 2, the investigative team will begin to develop a just-in-time adaptive mobile intervention, designed to promote 6-mercaptopurine adherence in this population, by developing contextually-tailored mobile messages and decision rules about the conditions under which to deliver the messages. Just-in-time mobile interventions are well-suited to address limitations of previous interventions by providing personalized adherence support, at the right time, only when it is needed. The creation of messages and decision rules will follow a rigorous mobile health development approach by iteratively incorporating self-management theory, empirical evidence, expert input, and stakeholder feedback. Focus groups with 15 AYA from Aim 1 will be conducted to user-test and refine the messages. In Aim 3, the just-in-time adaptive mobile intervention called AYA ADAPTS (Adherence Assessments and Personalized Timely Support) will be pilot tested in a micro-randomized trial with 30 newly recruited patients. AYA ADAPTS will integrate the tailored mobile messages and decision rules developed and refined in Aim 2. The research team will determine the feasibility and acceptability of this intervention in preparation for the PI's larger-scale optimization trial (R01 proposal). This proposal has methodological, theoretical, clinical, and technological innovations. The research and career development plan, supported by a multi-disciplinary team of experts in a rich academic environment, will support the PI's transition to an independent clinician-scientist who possesses the skills and expertise to use cutting-edge mobile health methods to design the most potent adherence- promotion interventions for pediatric cancer patients. This K08 will provide opportunities to acquire skills and knowledge in: (1) ecological momentary assessment and multi-level statistics, (2) behavior change intervention development applied to mobile health, and (3) the conduct of clinical trials and intervention dissemination and implementation.

PUBLIC HEALTH RELEVANCE: Stagnant survival and relapse rates for adolescents and young adults with cancer are partially attributed to a modifiable behavior—nonadherence to cancer treatments. To address multiple calls from the NCI and other federal agencies for research targeting disparities in cancer treatment outcomes for this population, this K08 research will use novel real time mobile health methodologies to examine and intervene on the time-varying, contextual factors that influence daily oral chemotherapy adherence in adolescents and young adults with acute lymphoblastic leukemia. The proposed research and career development plan will yield critical multi-level data and a just-in-time adaptive intervention for nonadherence, with the potential for the investigator to advance adherence intervention science and improve clinical outcomes in adolescents and young adults with cancer. !

CRITIQUE: The written critiques of individual reviewers are provided in essentially unedited form in this section. Please note that critiques and criteria scores, prepared prior to the review meeting, may not have been revised following discussions at the meeting. The "Resume and Summary of Discussion" section summarizes the final opinions of the review committee.

CRITIQUE 1

Candidate:	2
Career Development Plan/Career Goals /Plan to Provide Mentoring:	2
Research Plan:	3
Mentor(s), Co-Mentor(s), Consultant(s), Collaborator(s):	1
Environment Commitment to the Candidate:	1

Overall Impact: This is an initial K08 submission from Dr. Psihogios is a licensed clinical psychologist and postdoctoral fellow in the Division of Oncology at The Children's Hospital of Philadelphia (CHOP). She seeks additional training in ecological momentary assessment and multi-level statistics, behavior change intervention development incorporating mHealth, and clinical trials and intervention dissemination and implementation. The training plan and engagement of mentors is appropriate and well described. The planned research may be slightly ambitious, although the environment is outstanding. Enthusiasm is high for this clinically-relevant proposal that aims to address adherence to

6MP among adolescent and young adult cancer survivors diagnosed with acute lymphoblastic leukemia. Overall impact of this application is high on career development of the candidate.

1. Candidate:
Strengths
- Dr. Psihogios is a licensed clinical psychologist and postdoctoral fellow in the Division of Oncology at The Children's Hospital of Philadelphia (CHOP). She received her PhD from Loyola University in 2016.
- Assistant Professor-in-Residence of Medicine in the Division of Digestive and Liver Diseases and an Associate Member of the Prevention and Genetics Program at the Samuel Oschin Comprehensive Cancer Institute at Cedars-Sinai Medical Center.
- The candidate has 13 peer-reviewed publications, with 8 as first author.
- Dr. Psihogios is PI of an ACS Postdoctoral fellowship award and a Mattie Miracle Cancer Foundation award.

Weaknesses
- Dr. Psihogios is very early in her career; however, this concern is mitigated by her accomplishments to date.

2. Career Development Plan/Career Goals & Objectives:
Strengths
- Dr. Psihogios's long term goal is to become an independent clinician-scientist who improves cancer treatment outcomes in adolescents and young adults (AYA) by targeting nonadherence.
- The short-term goals including gaining skills and knowledge in (1) EMA and multi-level statistics applied to AYA with acute lymphoblastic leukemia (ALL), (2) behavior change intervention development incorporating mHealth to establish methodological competencies for developing effective and engaging adherence-promotion interventions, (3) clinical trials and intervention dissemination and implementation.
- The rationale, mentorship, coursework and seminars/workshops for each career goal are well delineated.

Weaknesses
- None noted.

3. Research Plan:
Strengths
- Use of a JITAI adaptive design is innovative and an appropriate method to evaluate adherence among AYA with ALL.
- The proposed work is guided by the Pediatric Self-Management Model and the mobile health tool development will be guided by the BUS Framework.
- Examples of the potential tailoring variables and mobile messages are provided.

Weaknesses
- The plans appear to be a bit ambitious in terms of developing the tailored messages, adapting the app to incorporate these messages and recruiting for the Aim 3 JITAI trial.

4. Mentor(s), Co-Mentor(s), Consultant(s), Collaborator(s):
Strengths
- The proposed mentoring team is very strong, led by Dr. Barakat, and includes Dr. Susan Murphy (adaptive interventions), Dr. Lisa Schwartz (AYA psycho-oncology), Dr. Linda Fleisher (development of mobile messages), Dr. Hunger (acute lymphoblastic leukemia), Dr. Laurenceau (statistics and ecological momentary assessment), Dr. Ahna Luise Hoff Pai (adherence).

Weaknesses
- None noted.

5. Environment and Institutional Commitment to the Candidate:
Strengths
- The environment is appropriate for the proposed work.

Weaknesses
- None noted.

Study Timeline
Strengths
- The timeline for the proposed research (and training activities) are well described.

Weaknesses
- None noted.

Protections for Human Subjects: Acceptable Risks and Adequate Protections.
- Risks, protections and benefits are well described by each aim.

Data and Safety Monitoring Plan (Applicable for Clinical Trials Only): Acceptable.

Inclusion of Women, Minorities and Children:
- Sex/Gender: Distribution justified scientifically.
- Race/Ethnicity: Distribution justified scientifically.
- For NIH-Defined Phase III trials, Plans for valid design and analysis: Scientifically acceptable.
- Inclusion/Exclusion of Children under 18: Including ages < 18; justified scientifically.
- Appropriate and well described.

Vertebrate Animals: Not Applicable (No Vertebrate Animals).

Biohazards: Not Applicable (No Biohazards).

Training in the Responsible Conduct of Research: Acceptable.
Comments on Format (Required):
- Online; in person.

Comments on Subject Matter (Required):
- Varied; appropriate.

Comments on Faculty Participation (Required; not applicable for mid- and senior-career awards):
- Interactions with mentors;

Comments on Duration (Required):
- Varies.

Comments on Frequency (Required):
- Varies.

Select Agents: Not Applicable (No Select Agents).

Resource Sharing Plans: Unacceptable.
- Not provided.

Authentication of Key Biological and/or Chemical Resources: Not Applicable (No Relevant Resources).

Budget and Period of Support: Recommend as Requested.

CRITIQUE 2

Candidate:	1
Career Development Plan/Career Goals /Plan to Provide Mentoring:	2
Research Plan:	2
Mentor(s), Co-Mentor(s), Consultant(s), Collaborator(s):	2
Environment Commitment to the Candidate:	2

Overall Impact: Dr. Alexandra Psihogios is a clinical-psychologist and a Postdoctoral Research Fellow in the Division of Oncology, CHOP, Philadelphia (2017-). The candidate's long-term goal is to become an independent clinician-scientist who improves cancer treatment outcomes in adolescents and young adults (AYA) by targeting non-adherence. To reach her research and career goal, she proposes a 5-yr K08 application. Dr. Psihogios is a stellar candidate with a strong commitment to cancer control particularly in the field of adherence intervention. The mentoring and advisory team are outstanding. The statements from the mentors clearly show their strong commitment to the candidate's success, describe the candidate's strengths and the areas needed improvements and also very clear description of the mentor's role in providing guidance and advice to achieve the candidate's career goal. The candidate also has strong institutional support and excellent research/ and training environment at CHOP-UPenn. The proposed study is innovative and well designed and has strong scientific premise. Overall impact is high.

1. Candidate:
Strengths
- The candidate has extensive training in pediatric psychology and an excellent track record of publications focused on children, adolescents, and young adults with a variety of chronic medical conditions (13 papers; 8 as first author and 2 book chapters). She also received numerous academic awards.
- The candidate has a high potential for becoming an independent investigator. She is a PI of several pilot grants. Strong reference letters indicating this as well.
- Strong motivation and commitment to meeting the program objectives to become an independent investigator in research.

Weaknesses
- No major weaknesses noted.

2. Career Development Plan/Career Goals & Objectives:
Strengths
- The candidate's prior training and research experience are appropriate for this award.
- Very clear description of the plans for monitoring and evaluating the candidate's progress toward independence.
- Nice integration of research activities, training activities with candidate's research and career goals.

Weaknesses
- None noted.

3. Research Plan:
Strengths
- The proposed study is innovative and supported with a strong scientific premise.
- Study is well-designed with alternative strategies provided for potential pitfalls.
- The proposed research is appropriate to the candidate's stage of research development and can provide her the research skills necessary as described in the CDP.

Weaknesses
- The research plan is somewhat ambitious, however, this is not a major concern because of the promising candidate and strong mentorship.

4. Mentor(s), Co-Mentor(s), Consultant(s), Collaborator(s):
Strengths
- Mentoring committee and advisory committee have relevant and complementary research experience.
- The primary mentor Dr. Lamia Barakat is a well-known researcher in psycho-oncology, and co-mentors Dr. Susan Murphy is an expert on mobile health and Dr. Lisa Schwartz is an AYA oncology researcher.
- Dr. Barakat has an excellent mentoring experience and is suitable to provide mentorship in behavior intervention development and the conduct of clinical trials in the context of pediatric oncology (in addition to the mentoring and advisory committee). This is an area that was not provided in the candidate's postdoctoral training.
- Very clear description of the quality and extent of the mentors' proposed role in providing guidance and advice to the candidate and also for plans for monitoring and evaluating the candidate's progress toward independence.

Weaknesses
- None noted.

5. Environment and Institutional Commitment to the Candidate:
Strengths
- The Division of Oncology is fully committed to protecting 75% of the candidate's time for CDP activities. 25% will be dedicated to clinical practice in the cancer center.
- Committed to promote the candidate to an Instructor in Pediatrics in June 2019, not contingent on receiving K08 award.
- CHOP-UPENN environment are ideal for the candidate's proposed training and research.

Weaknesses
- No major weaknesses noted.

Study Timeline
Strengths
- Study timeline for study aims is described in detail including the feasibility of research activities.

Weaknesses
- None noted.

Protections for Human Subjects: Acceptable Risks and Adequate Protections.

Data and Safety Monitoring Plan (Applicable for Clinical Trials Only):

Inclusion of Women, Minorities and Children:
- Sex/Gender: Distribution justified scientifically.
- Race/Ethnicity: Distribution justified scientifically.
- For NIH-Defined Phase III trials, Plans for valid design and analysis: Not applicable.
- Inclusion/Exclusion of Children under 18: Including ages <18; not justified scientifically

Vertebrate Animals: Not Applicable (No Vertebrate Animals).

Biohazards: Not Applicable (No Biohazards).

Training in the Responsible Conduct of Research: Acceptable.

Select Agents: Not Applicable (No Select Agents).

Resource Sharing Plans: Acceptable.

Authentication of Key Biological and/or Chemical Resources: Not Applicable (No Relevant Resources).

Budget and Period of Support: Recommend as Requested.

CRITIQUE 3

Candidate:	2
Career Development Plan/Career Goals /Plan to Provide Mentoring:	4
Research Plan:	5
Mentor(s), Co-Mentor(s), Consultant(s), Collaborator(s):	1
Environment Commitment to the Candidate:	1

Overall Impact: Dr. Psihogios is a clinical psychologist completing her post-doctoral training at Children's Hospital of Pennsylvania. She seeks to leverage effective interventions that improve cancer treatment adherence in real-time among adolescent and young adult cancer patients diagnosed with acute lymphoblastic leukemia (ALL). Dr. Psihogios seeks to employ ecological momentary assessment (EMA) methodologies to obtain contextual, mood/affect and social (peer/family), physical symptoms and motivation level data from brief smartphone-based surveys combined with adherence data collected from MEMS caps. Dr. Psihogios has established a well-rounded team of mentors to provide overview and support for the proposed training and research activities. However, the training plan could be strengthened with the addition of more formal coursework over the entire CDA period. Finally, there are data collection, transfer and management and safety considerations that need to be acknowledged in the application of EMA in the proposed work.

1. Candidate:
Strengths
- Dr. Psihogios is a licensed clinical psychologist and a post-doctoral fellow at the Children's Hospital of Pennsylvania. She has completed a MA and PhD in Clinical Psychology at Loyola University and a pre-doctoral clinical internship in pediatric psychology at Children's Hospital of Philadelphia.
- Dr. Psihogios has received 2 grants to fund pilot studies on medication adherence among AYA cancer patients and a text messaging service to assess oral chemotherapy adherence.
- A solid publication record that includes 13 peer-reviewed articles (8 as first author).
- Two of these publications deal with childhood/AYA cancer.

Weaknesses
- No major weakness noted.

2. Career Development Plan/Career Goals & Objectives:
Strengths
- Dr. Psihogios seeks to gain further training in novel and timely intervention mechanisms that are relevant to AYA cancer patients by pursuing the following training goals (1) Ecological momentary assessment (EMA) and multi-level statistics applied to AYA with acute lymphoblastic leukemia (ALL) who are at-risk for nonadherence, (2) behavior change intervention development incorporating mHealth in effective adherence-promotion interventions, (3) clinical trials and intervention dissemination and implementation.

Weaknesses
- Training with Dr. Schwartz is in recruitment/retention with AYA oncology patients, but formal course work in Cancer and Health Disparities (even beyond seminars in pediatric oncology) is recommended.

- Dr. Psihogios proposes 1 course per year for years 1-3 and none in years 4-5; would suggest more formal, didactic coursework rather than weekly meetings/tutorials in advanced survey, study design and statistical methods to take advantage of this 5-year training period. Also consider training programs offered by NIH, such as OBSSR's Clinical Trials summer institute and other relevant trainings.

3. Research Plan:
Strengths
- Dr. Psihogios seeks to evaluate a potentially novel and timely adherence intervention for AYA cancer patients diagnosed with acute lymphoblastic leukemia. She seeks to undertake the following research activities: (1) to conduct a 6-month EMA study with 30 AYA with ALL in the maintenance phase of treatment and their caregivers to determine the temporal associations between intrapersonal and interpersonal contextual variables and electronically-monitored 6MP adherence; (2) develop contextually-tailored mobile messages and potential decision rules for a JITAI; and (3) pilot test the JITAI in a 28-day micro-randomized trial.
- This theoretically grounded and highly innovative intervention has the potential to meet the needs of AYA cancer patients and enhance medication adherence outcomes.

Weaknesses
- Will RAs review how to enter EMA data to both AYA and their caregivers? Will the app be available for both Apple and android (to name just two, but there are other devises) iOS (as well as other operating systems)? How and when will data be transferred to CHOP?
- EMA data will first be collected on smartphones; will they be stored there as well? How and when will data be transferred to CHOP? Is there a secure server that data can be transferred to? Will patients be assigned unique IDs to ensure that data transfers are confidential? Please provide information on data back-up plans and also a contingency plan for storing data on smartphones in case transfers are not possible. Information on maintaining data security is required. Can patient delay their responses? What if they turn off their phones?

4. Mentor(s), Co-Mentor(s), Consultant(s), Collaborator(s):
Strengths
- Dr. Psihogios has identified a primary mentor (Dr. Lamia Barakat), two co-mentors, (Drs. Susan Murphy and Lisa Schwartz) and four content advisors (Drs. Jean-Phillipe Laurenceau, Ahna Pai, Linda Fleisher, and Stephen Hunger).
- Dr. Barakat provides expertise in psycho-oncology and behavior change interventions in pediatric populations. Dr. Murphy provides expertise in mHealth interventions and JITAI. Dr. Schwartz provides mentorship in AYA cancer. The advisory team provides collective expertise in EMA and statistical analysis of EMA data (Laurenceau), adherence and pediatric oncology (Pai), health communications (Fleisher) and pediatric ALL (Fleisher).

Weaknesses
- None noted.

5. Environment and Institutional Commitment to the Candidate:
Strengths
- Children's Hospital of Philadelphia (CHOP) is an excellent environment to conduct the proposed activities.
- The applicant has a commitment from the Division of Oncology for 75% protected time for scholarly activity.
- Dr. Hunger, Division Chief of Pediatric Oncology, indicates that they have initiated the process of appointing Dr. Psihogios as an Instructor in Pediatrics, to begin in June 2019 after she completes her fellowship.

Weaknesses
- None noted.

Study Timeline
Strengths
- Study timeline for each study aim is included and appropriately describes feasibility of study activities.

Weaknesses
- None noted.

Protections for Human Subjects: Acceptable Risks and Adequate Protections.
- Description of risks and adequate protection against risks is appropriately described.

Data and Safety Monitoring Plan (Applicable for Clinical Trials Only): Acceptable.

Inclusion of Women, Minorities and Children:
- Sex/Gender: Distribution justified scientifically.
- Race/Ethnicity: Distribution justified scientifically.
- For NIH-Defined Phase III trials, Plans for valid design and analysis: Not applicable.
- Inclusion/Exclusion of Children under 18: Including ages < 18; justified scientifically.
- All sex/gender, race/ethnicity, and age-based inclusion/exclusion criteria are scientifically. justified.

Vertebrate Animals: Not Applicable (No Vertebrate Animals).

Biohazards: Not Applicable (No Biohazards).

Training in the Responsible Conduct of Research: Acceptable.

Select Agents: Not Applicable (No Select Agents).

Resource Sharing Plans: Acceptable.

Authentication of Key Biological and/or Chemical Resources: Not Applicable (No Relevant Resources).

Budget and Period of Support: Recommend as Requested.

THE FOLLOWING SECTIONS WERE PREPARED BY THE SCIENTIFIC REVIEW OFFICER TO SUMMARIZE THE OUTCOME OF DISCUSSIONS OF THE REVIEW COMMITTEE, OR REVIEWERS' WRITTEN CRITIQUES, ON THE FOLLOWING ISSUES:

PROTECTION OF HUMAN SUBJECTS: ACCEPTABLE

INCLUSION OF WOMEN PLAN: ACCEPTABLE

INCLUSION OF MINORITIES PLAN: ACCEPTABLE

INCLUSION OF CHILDREN PLAN: ACCEPTABLE

COMMITTEE BUDGET RECOMMENDATIONS: The budget was recommended as requested.

Footnotes for 1 K08 CA241335-01; PI Name: Psihogios, Alexandra

NIH has modified its policy regarding the receipt of resubmissions (amended applications). See Guide Notice NOT-OD-14-074 at http://grants.nih.gov/grants/guide/notice-files/NOT-OD-14-074.html. The impact/priority score is calculated after discussion of an application by averaging the overall scores (1-9) given by all voting reviewers on the committee and multiplying by 10. The criterion scores are submitted prior to the meeting by the individual reviewers assigned to an application, and are not discussed specifically at the review meeting or calculated into the overall impact score. Some applications also receive a percentile ranking. For details on the review process, see http://grants.nih.gov/grants/peer_review_process.htm#scoring.

MEETING ROSTER

Subcommittee J - Career Development
National Cancer Institute Initial Review Group
NATIONAL CANCER INSTITUTE
NCI-J
02/28/2019 - 03/01/2019

Notice of NIH Policy to All Applicants: Meeting rosters are provided for information purposes only. Applicant investigators and institutional officials must not communicate directly with study section members about an application before or after the review. Failure to observe this policy will create a serious breach of integrity in the peer review process, and may lead to actions outlined in NOT-OD-14-073 at https://grants.nih.gov/grants/guide/notice-files/NOT-OD-14-073.html and NOT-OD-15-106 at https://grants.nih.gov/grants/guide/notice-files/NOT-OD-15-106.html, including removal of the application from immediate review.

CHAIRPERSON(S)

EPPLEIN, MEIRA, PHD
ASSOCIATE PROFESSOR
DEPARTMENT OF POPULATION HEALTH SCIENCES
CO-LEADER, CANCER CONTROL AND POPULATION SCIENCES
DUKE CANCER INSTITUTE
DUKE UNIVERSITY SCHOOL OF MEDICINE
DURHAM, NC 27705

GRAVES, KRISTI D., PHD
ASSOCIATE PROFESSOR
DEPARTMENT OF ONCOLOGY
CANCER PREVENTION AND CONTROL PROGRAM
LOMBARDI COMPREHENSIVE CANCER CENTER
GEORGETOWN UNIVERSITY
WASHINGTON, DC 20007

MEMBERS

AGARWAL, RAJESH, PHD
PROFESSOR AND VICE CHAIRMAN, DEPARTMENT OF PHARMACEUTICAL SCIENCES
CANCER PREVENTION AND CONTROL PROGRAM
UNIVERSITY OF COLORADO SKAGGS
SCHOOL OF PHARMACY AND PHARMACEUTICAL SCIENCES
UNIVERSITY OF COLORADO CANCER CENTER
AURORA, CO 80045

AHMAD, NIHAL, PHD *
PROFESSOR
DEPARTMENT OF DERMATOLOGY
SCHOOL OF MEDICINE AND PUBLIC HEALTH
UNIVERSITY OF WISCONSIN-MADISON
MADISON, WI 53706

BASU, ALAKANANDA, PHD *
PROFESSOR
DEPARTMENT OF MOLECULAR BIOLOGY AND IMMUNOLOGY
HEALTH SCIENCE CENTER
UNIVERSITY OF NORTH TEXAS
FORT WORTH, TX 76107

CHOI, JAEHYUK, MD, PHD *
ASSISTANT PROFESSOR
DEPARTMENT OF DERMATOLOGY
NORTHWESTERN UNIVERSITY
CHICAGO, IL 60611

DAI, MUSHUI, PHD *
ASSOCIATE PROFESSOR
DEPARTMENT OF MOLECULAR AND MEDICAL GENETICS
CO-DIRECTOR OF GRADUATE STUDIES
SCHOOL OF MEDICINE
OREGON HEALTH & SCIENCES UNIVERSITY
PORTLAND, OR 97239

DENG, YIBIN, MD, PHD *
ASSOCIATE PROFESSOR
DEPARTMENT OF CANCER GENETICS
UNIVERSITY OF MINNESOTA HORMEL INSTITUTE
AUSTIN, MN 55912

DIXON, DAN ALAN, PHD
ASSOCIATE PROFESSOR
DEPARTMENT OF MOLECULAR BIOSCIENCES
CO-LEADER, CANCER PREVENTION, SURVIVORSHIP PROGRAM
UNIVERSITY OF KANSAS CANCER CENTER
UNIVERSITY OF KANSAS
KANSAS CITY, KS 66160

DUDLEY, ANDREW CARL, PHD *
ASSOCIATE PROFESSOR
DEPARTMENT OF MICROBIOLOGY, IMMUNOLOGY AND CANCER BIOLOGY
EMILY COURIC CANCER CENTER
UNIVERSITY OF VIRGINIA
CHARLOTTESVILLE, VA 22908

GANEM, NEIL J., PHD
ASSISTANT PROFESSOR
DIVISION OF HEMATOLOGY AND ONCOLOGY
DEPARTMENT OF PHARMACOLOGY AND EXPERIMENTAL THERAPEUTICS
BOSTON UNIVERSITY SCHOOL OF MEDICINE
BOSTON, MA 02118

GOLEMIS, ERICA A., PHD
PROFESSOR AND SENIOR MEMBER
DEPUTY CHIEF SCIENTIFIC OFFICER
DEPARTMENT OF DEVELOPMENTAL THERAPEUTICS
FOX CHASE CANCER CENTER
PHILADELPHIA, PA 19111

HATCHER, JENNIFER, PHD
PROFESSOR
COLLEGE OF PUBLIC HEALTH
UNIVERSITY OF ARIZONA
PHOENIX, AZ 85004

HAWSE, JOHN R, PHD *
ASSOCIATE PROFESSOR
DEPARTMENT OF BIOCHEMISTRY AND MOLECULAR BIOLOGY
MAYO CLINIC
ROCHESTER, MN 55905

JAIN, MANEESH, PHD
ASSOCIATE PROFESSOR
DEPARTMENT OF BIOCHEMISTRY AND MOLECULAR BIOLOGY
COLLEGE OF MEDICINE
FRED AND PAMELA BUFFETT CANCER CENTER
UNIVERSITY OF NEBRASKA MEDICAL CENTER
OMAHA, NE 68198

KAHALLEY, LISA SCHUM, PHD
ASSOCIATE PROFESSOR
DEPARTMENT OF PEDIATRICS
SECTION OF PSYCHOLOGY
BAYLOR COLLEGE OF MEDICINE
TEXAS CHILDREN'S HOSPITAL
HOUSTON, TX 77030-2399

KAPADIA, FARZANA, PHD *
ASSOCIATE PROFESSOR OF GLOBAL PUBLIC HEALTH & POPULATION HEALTH
DEPARTMENT OF EPIDEMIOLOGY
COLLEGE OF GLOBAL PUBLIC HEALTH
NEW YORK UNIVERSITY
NEW YORK, NY 10003

KROGSGAARD, MICHELLE, PHD
ASSOCIATE PROFESSOR
DEPARTMENT OF PATHOLOGY
NEW YORK UNIVERSITY SCHOOL OF MEDICINE
NEW YORK, NY 10016

KRUPNICK, ALEXANDER S., MD
ASSOCIATE PROFESSOR OF SURGERY
SURGICAL DIRECTOR OF LUNG TRANSPLANTATION
DEPARTMENT OF SURGERY
DIVISION OF THORACIC SURGERY
UNIVERSITY OF VIRGINIA
CHARLOTTESVILLE, VA 22908

LACORAZZA, DANIEL, PHD *
ASSOCIATE PROFESSOR
DEPARTMENT OF PATHOLOGY AND IMMUNOLOGY
TEXAS CHILDREN'S HOSPITAL
BAYLOR COLLEGE OF MEDICINE
HOUSTON, TX 77030

LAI, ALBERT, MD, PHD *
PROFESSOR
DEPARTMENT OF NEUROLOGY
UCLA NEURO-ONCOLOGY PROGRAM
UNIVERSITY OF CALIFORNIA, LOS ANGELES
LOS ANGELES, CA 90095

LO, HUI-WEN, PHD
PROFESSOR OF CANCER BIOLOGY
DEPARTMENT OF CANCER BIOLOGY
WAKE FOREST UNIVERSITY SCHOOL OF MEDICINE
WAKE FOREST COMPREHENSIVE CANCER CENTER
WINSTON-SALEM, NC 27157

PAGEL, JOHN M., MD, PHD
CHIEF OF HEMATOLOGIC MALIGNANCIES PROGRAM
DIRECTOR, STEM CELL TRANSPLANTATION
SWEDISH MEDICAL CENTER
SEATTLE, WA 98104

RUELLA, MARCO, MD *
ASSISTANT PROFESSOR OF MEDICINE
SCIENTIFIC DIRECTOR LYMPHOMA PROGAM
CENTER FOR CELLULAR IMMUNOTHERAPIES
PERELMAN CENTER FOR ADVANCED MEDICINE
UNIVERSITY OF PENNSYLVANIA
PHILADELPHIA, PA 191004

SETIAWAN, VERONICA WENDY, PHD
ASSOCIATE PROFESSOR
DEPARTMENT OF PREVENTIVE MEDICINE
KECK SCHOOL OF MEDICINE
NORRIS COMPREHENSIVE CANCER CENTER
UNIVERSITY OF SOUTHERN CALIFORNIA
LOS ANGELES, CA 90033

TESSEMA, MATHEWOS, DVM, PHD *
ASSOCIATE SCIENTIST
LUNG CANCER PROGRAM
LOVELACE RESPIRATORY RESEARCH INSTITUTE
ALBUQUERQUE, NM 87108

TORRES-ROCA, JAVIER F, MD *
ASSOCIATE MEMBER
OF RADIATION ONCOLOGY
H. LEE MOFFIT CANCER CENTER
ASSOCIATE PROFESSOR OF ONCOLOGIC SCIENCES,
UNIVERSITY OF SOUTH FLORIDA
TAMPA, FL 33612

TRAN, DAVID D, MD, PHD *
ASSISTANT PROFESSOR
LILLIAN S. WELLS DEPARTMENT OF NEUROSURGERY
UNIVERSITY OF FLORIDA
GAINESVILLE, FL 32610

WU, JIE, PHD
PROFESSOR, PEGGY AND CHARLES STEPHENSON CHAIR IN CANCER TRANSLATIONAL RESEARCH
DEPARTMENT OF PATHOLOGY
PEGGY AND CHARLES STEPHENSON CANCER CENTER
UNIVERSITY OF OKLAHOMA HEALTH SCIENCES CENTER
OKLAHOMA CITY, OK 73104

ZHENG, LEI, MD, PHD
ASSOCIATE PROFESSOR
DEPARTMENT OF ONCOLOGY
THE SIDNEY KIMMEL COMPREHENSIVE CANCER CENTER
JOHNS HOPKINS UNIVERSITY
BALTIMORE, MD 21231

ZHOU, GANG, PHD
ASSOCIATE PROFESSOR, CANCER IMMUNOLOGY,
INFLAMMATION AND TOLERANCE PROGRAM
GEORGIA CANCER CENTER
COLLEGE OF GRADUATE STUDIES
AUGUSTA UNIVERSITY
AUGUSTA , GA 30912

SCIENTIFIC REVIEW OFFICER

DEB, TUSHAR, PHD
SCIENTIFIC REVIEW OFFICER
RESOURCES & TRAINING REVIEW BRANCH
DIVISION OF EXTRAMURAL ACTIVITIES
NATIONAL CANCER INSTITUTE
NATIONAL INSTITUTES OF HEALTH
ROCKVILLE, MD 20850

EXTRAMURAL SUPPORT ASSISTANT

WILSON, BRIDGETTE
EXTRAMURAL SUPPORT ASSISTANT
RESOURCE & TRAINING REVIEW BRANCH
DIVISION OF EXTRAMURAL ACTIVITIES
NATIONAL CANCER INSTITUTE- SHADY GROVE
NATIONAL INSTITUTES OF HEALTH
BETHESDA, MD 20892

PROGRAM REPRESENTATIVE

LIM, SUSAN E, PHD
PROGRAM DIRECTOR
CANCER TRAINING BRANCH
CENTER FOR CANCER TRAINING
NATIONAL CANCER INSTITUTE
NATIONAL INSTITUTES OF HEALTH
ROCKVILLE, MD 20850

OJEIFO, JOHN O, MBBS, PHD
PROGRAM DIRECTOR
DIVERSITY TRAINING BRANCH
CENTER TO REDUCE CANCER HEALTH DISPARITIES
NATIONAL CANCER INSTITUTE
NATIONAL INSTITUTES OF HEALTH
ROCKVILLE, MD 20850

RADAEV, SERGEI, PHD
PROGRAM DIRECTOR
OFFICE OF CANCER CENTERS
NATIONAL CANCER INSTITUTE
NATIONAL INSTITUTE OF HEALTH
BETHESDA, MD 20892

SOYOMBO-SHOOLA, ABIGAIL ADEBISI, PHD
PROGRAM DIRECTOR
DIVERSITY TRAINING BRANCH
CENTER TO REDUCE CANCER HEALTH DISPARTITIES
NATIONAL CANCER INSTITUTE
NATIONAL INSTITUTES OF HEALTH
ROCKVILLE, MD 20850

* Temporary Member. For grant applications, temporary members may participate in the entire meeting or may review only selected applications as needed.

Consultants are required to absent themselves from the room during the review of any application if their presence would constitute or appear to constitute a conflict of interest.
